# Development of robust machine learning models to estimate hydrochar higher heating value and yield based upon biomass proximate analysis

**DOI:** 10.1186/s40643-025-00979-1

**Published:** 2025-12-03

**Authors:** Guoliang Hou, Ahmad Alkhayyat, Ahmad Almalkawi, Anupam Yadav, H. S. Shreenidhi, Vishnu Saini, Shirin Shomurotova, Devendra Singh, Vatsal Jain, Aseel Smerat, Ahmad Khalid

**Affiliations:** 1 School of Mathematics, Changchun Normal University, Changchun, 130032 Jilin China; 2 Department of Computers Techniques Engineering, College of Technical Engineering, The Islamic University, Najaf, Iraq; 3 Center for ESL & Academic Preparation, Modern College of Business and Science, Muscat, Oman; 4 Department of Computer Engineering and Application, GLA University, Mathura, 281406 India; 5 Department of Computer Science and Engineering, School of Engineering and Technology, JAIN (Deemed to Be University), Bangalore, Karnataka India; 6 Sharda School of Engineering and Sciences, Sharda University, Knowledge Park III, Greater Noida, 201310 Uttar Pradesh India; 7 Department of Chemistry Teaching Methods, National Pedagogical University of Uzbekistan, Bunyodkor Street 27, Tashkent, Uzbekistan; 8 Department of Computer Science & Engineering, Uttaranchal Institute of Technology, Uttaranchal University, Dehradun, Uttarakhand 248007 India; 9 Centre for Research Impact & Outcome, Chitkara University Institute of Engineering and Technology, Chitkara University, Rajpura, Punjab 140401 India; 10 Faculty of Educational Sciences, Al-Ahliyya Amman University, Amman, 19328 Jordan; 11 Department of Biosciences, Saveetha School of Engineering, Saveetha Institute of Medical and Technical Sciences, Chennai, 602105 India; 12 Faculty of Engineering, Sana’a University, Sanaa, Yemen

**Keywords:** Biomass proximate analysis, Hydrochar yield prediction, Machine learning, Higher heating value (HHV), CatBoost algorithm

## Abstract

This study introduces a robust machine learning framework for predicting hydrochar yield and higher heating value (HHV) using biomass proximate analysis. A curated dataset of 481 samples was assembled, featuring input variables such as fixed carbon, volatile matter, ash content, reaction time, temperature, and water content. Hydrochar yield and HHV served as the target outputs. To enhance data quality, Monte Carlo Outlier Detection (MCOD) was employed to eliminate anomalous entries. Thirteen machine learning algorithms, including convolutional neural networks (CNN), linear regression, decision trees, and advanced ensemble methods (CatBoost, LightGBM, XGBoost) were systematically compared. CatBoost demonstrated superior performance, achieving an R^2^ of 0.98 and mean squared error (MSE) of 0.05 for HHV prediction, and an R^2^ of 0.94 with MSE of 0.03 for yield estimation. SHAP analysis identified ash content as the most influential feature for HHV prediction, while temperature, water content, and fixed carbon were key drivers of yield. These results validate the effectiveness of gradient boosting models, particularly CatBoost, in accurately modeling hydrothermal carbonization outcomes and supporting data-driven biomass valorization strategies.

**Graphical abstract:**

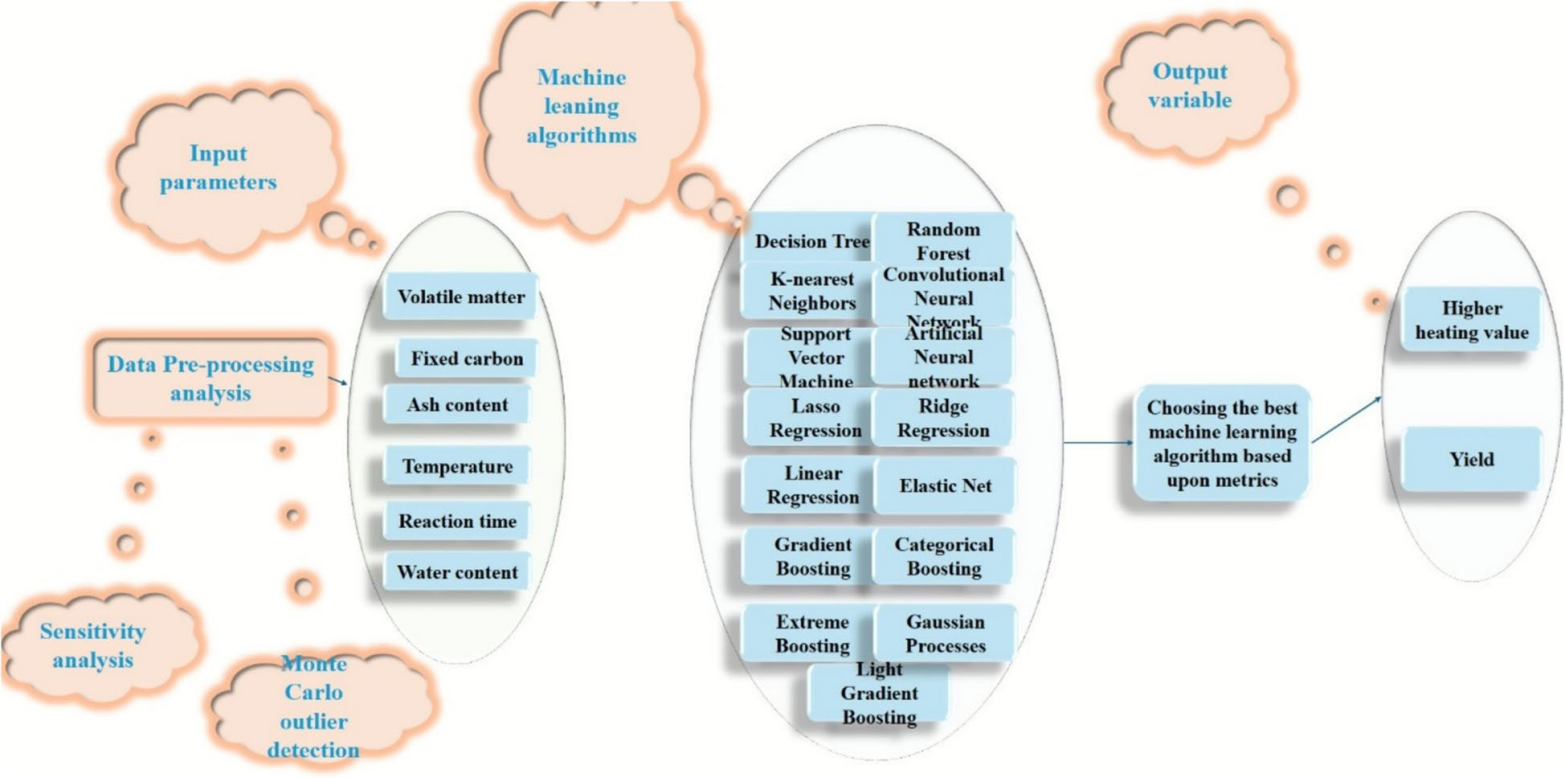

**Supplementary Information:**

The online version contains supplementary material available at 10.1186/s40643-025-00979-1.

## Introduction

The majority of chemical requirements and global energy are presently satisfied by fossil fuels. In light of the declining availability of fossil reserves and their adversative environmental consequences, such as air pollution and climate change, scientists are exploring justifiable alternatives for energy production (Yang et al. 2022; Aghdam et al. 2023; Khezerlooe-ye Aghdam et al. 2019; Li 2025; Wu et al. 2025; Yu et al. 2023). Carbon–neutral biomass energy has appeared as a prominent focus among renewable energy resources over recent decades (Chen et al. 2022a; Wang et al. 2025; Xu et al. 2024) primarily because it can be transformed into fossil-like liquid, gaseous, and solid fuels with versatile applications. As the most significant renewable source of energy on Earth, bioenergy constitutes roughly 10 percent of the worldwide key energy supply (Tauro et al. 2021; Li et al. 2025; Niu et al. 2022; Dou et al. 2025). Approximately semi of worldwide biomass usage is dedicated to cooking and heating in emergent nations (Bhutto et al. 2019). To address this, shifting from traditional biomass to advanced modern energy solutions like biodiesel, biogas, biopower, and bioethanol is essential to minimize adverse environmental effects.

Several thermochemical technologies have been proposed to change biomass into biochemicals and biofuels (Chen et al. 2022b). Thermochemical manners, such as pyrolysis, gasification, combustion, and hydrothermal carbonization (HTC), have drawn substantial scientific interest (Hai et al. 2023; Paula et al. 2022). These approaches allow the straight conversion of biomass into value-added products without the need for chemically harsh and energy-intensive pretreatment procedures. However, the high content of moisture for many biomass feedstocks, both dedicated and waste-derived, renders them inappropriate for certain thermochemical pathways like pyrolysis, combustion, and gasification (Li 2025; Shafizadeh et al. 2022; Tian et al. 2025). Current methods are inefficient at handling wet biomass, resulting in substantial expenses for humidity decrease. Hydrothermal processing technologies, with HTC at the forefront, present a practical and cost-effective solution by eliminating the energy-intensive drying phase. Beyond this, HTC is also more energy-efficient than other thermal conversion processes like gasification and pyrolysis (Zhang et al. 2019) and, due to its slighter reaction circumstances, generates minimum amounts of toxic gases, like sulfur oxides and nitrogen.

The hydrothermal carbonization (HTC) procedure is conducted in pressed water at 180 and 260 °C, mimicking the ordinary coalification development that happened over millions of years. This progression produces a carbon-rich known as hydrochar (Xu et al. 2020), Hydrochar’s properties make it comparable to lignite. Biochar which formed in pyrolysis, is specifically suited for submissions for instance, water treatment, carbon sequestration, and agriculture due to its large carbon content, reduced reactivity, and high surface area (Xiong et al. 2019). Hydrochar has higher ash content, lower carbon content, and greater reactivity (Xu et al. 2021; Shi et al. 2021), positioning it as an interesting compound for soil amendment, energy generation, and pollutant adsorption (Li et al. 2025). Generated under comparatively mild circumstances in comparison to biochar, hydrochar demonstrates reduced porosity, lower stability, lower pH, and smaller specific surface area (Liu et al. 2022). However, its abundance of oxygenated groups, containing carbonyl, carboxyl and phenolic hydroxyl, enhances its ability to adsorb metallic element (Tsarpali et al. 2022).

The structural and physico-chemical properties of hydrochar, highly depend on the nature of the biomass and the parameters of the HTC. Detection of the ideal treating conditions for a given biomass feedstock typically involves many laboratory tests, which are resource-intensive, laborious, and expensive. These restrictions hinder the detailed characterization of hydrochar’s quantity and quality. The inherent complexity of interactions and mechanisms during the hydrothermal carbonization (HTC) process renders current computational techniques inadequate for accurately predicting hydrochar properties. Consequently, it is imperative to develop more effective and precise methodologies for assessing the structural and physicochemical characteristics of hydrochar, thereby ensuring its appropriate utilization across various applications (Shafizadeh et al. 2023; Fang et al. 2025; Xu and Liang 2025; Yang et al. 2025; Zhang et al. 2025).

**Fig. 1:**
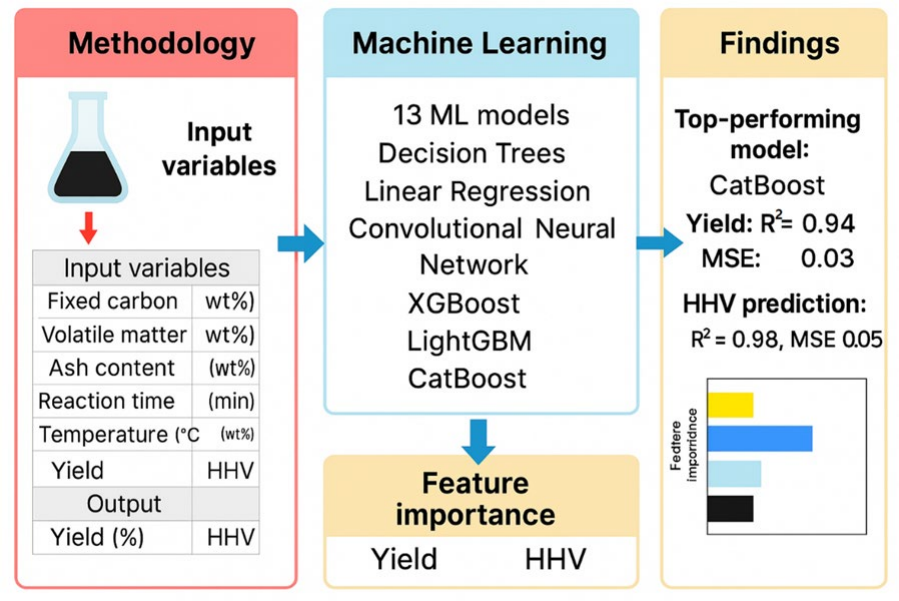
Overall workflow taken in this study to construct the data-driven models and choose the top-performing one subsequently

This work presents a novel and rigorous methodology for predicting hydrochar’s HHV and yield. Our primary contribution is the systematic evaluation of a broad spectrum of machine learning algorithms, including traditional models (linear regression, SVM) and advanced ensemble and deep learning techniques (ANN, CNN, RF, XGBoost, CatBoost, LightGBM). This comprehensive head-to-head performance comparison sets a new benchmark in this research area. We also introduce a robust Monte Carlo-based outlier detection to enhance dataset reliability, a step often overlooked in similar studies. To ensure the trustworthiness of our findings, we provide a detailed analysis of model performance using multiple metrics and graphical representations. The final novelty of our work is the application of SHAP analysis on the best-performing model, which not only validates our results but also provides crucial insights into the underlying relationships between biomass composition and hydrochar properties, thereby advancing the fundamental understanding of this process. The complete methodology context is illustrated in Fig. 1.

## Machine learning backgrounds

This segment details the machine learning algorithms.

### Convolutional neural network

It is a specialized class of algorithms planned to process structured data, such as images, by leveraging their spatial hierarchies. Inspired by the visual cortex of the human brain, CNNs utilize a blend of pooling, convolutional, and fully connected layers to detect and learn features at different levels of complexity. Convolutional layers use filters to recognize patterns like textures and edges, while pooling layers decrease dimensionality, increasing computational efficiency and resilience to input variations. Fully connected layers integrate these features to produce final predictions. The training process, driven by backpropagation, adjusts the network’s weights to minimize prediction error, while activation functions such as ReLU introduce non-linearity to capture complex relationships in the data.

CNNs excel in a variety of computer vision tasks, including image classification, object detection, and face recognition, where their ability to extract features automatically makes them highly effective. Beyond traditional vision applications, CNNs have been extended to other fields as well. In medical imaging, they assist in disease diagnosis by analyzing features in scans or X-rays. In e-commerce and video analytics, CNNs are employed for tasks such as optical character recognition (OCR) and automated tagging. Even in autonomous driving, CNNs play a vital role in lane detection and obstacle avoidance, while adaptations of CNNs in natural language processing (NLP) are used for sequence modeling and text analysis. Their adaptability across domains makes them indispensable tools in modern artificial intelligence.

Despite their advantages, CNNs come with challenges. They require large labeled datasets and substantial computational resources for training, which can be a limiting factor in many applications. CNNs are also susceptible to adversarial attacks, where subtle changes to input data can mislead predictions. Moreover, overfitting is a common issue, particularly when training with limited data, necessitating regularization techniques and hyperparameter optimization to achieve strong generalization (Li et al. 2022; Kim 2017; Lopez Pinaya, et al. 2020; Aloysius and Geetha 2017; Chagas, et al. 2018).

### Artificial neural network

ANNs are computational manners encouraged by biological neural systems consisting of interconnected layers of neurons. Data is processed through these layers, with each connection having a weight that is adjusted during learning to emphasize or de-emphasize certain features. This learning process is driven by backpropagation, where the loss function’s gradient is computed and used to update the weights iteratively. This enables ANNs to progress their ability to model complex patterns progressively (Fan et al. 2025; Yue 2025; Zhang et al. 2017).

ANNs are highly versatile and can be applied across various fields. In healthcare, they are used for medical diagnostics, imaging, or genomics data to detect disease patterns. In finance, ANNs assist in credit scoring and risk assessment, leveraging historical data to predict outcomes. They are also used in marketing for customer segmentation and behavior analysis, in natural language processing for tasks like sentiment analysis, and robotics for autonomous decision-making.

Despite their advantages, ANNs have challenges. They need large data and substantial computational power to train, and their training can be time-consuming. Additionally, the “black-box” nature of ANNs makes interpreting their decision-making processes difficult, which is problematic in areas requiring transparency, such as healthcare or legal systems. Nonetheless, ANNs remain powerful tools for tasks involving complex data (Khan, et al. 2019; Ahmadi et al. 2013; Gardner and Dorling 1998; Zhu 2022; Heidari et al. 2016).

### Decision tree

A Decision Tree is a flowchart-like used in machine learning, where each internal node characterizes a test on a feature, and branches represent possible outcomes. Leaf nodes indicate the final decision or classification. The tree splits the data based on criteria such as information gain, Gini impurity, or variance reduction, targeting to generate subsets that are as homogeneous as possible.

The tree starts with the root node demonstrating the entire data. Then, it splits the data into smaller subsets based on feature values, optimizing the separation of classes or minimizing variance in regression. The feature that most effectively splits the data, i.e., providing the highest information gain or variance reduction, tends to be placed near the root. This process continues until stopping conditions are met.

Decision Trees are valued for their interpretability and simplicity. The structure lets users to trace the path from the root to the leaf to understand the decision-making process. This makes them suitable for applications like medical diagnostics, where transparency is critical, or in finance for tasks like credit scoring.

However, Decision Trees are prone to overfitting, especially with complex trees. This can be mitigated with pruning or ensemble techniques such as Random Forests or Gradient Boosting. Despite their limitations, Decision Trees remain a influential tool for various practical machine-learning tasks due to their clarity and ease of use (Ghiasi et al. 2020; Hautaniemi et al. 2005).

### Random forest

Random Forest is a technique that generates multiple decision trees during training and aggregates their predictions to enhance model precision and robustness. This method, based on bagging (bootstrap aggregating), involves training several models on various random subsets of the original dataset drawn with replacement. By averaging predictions in regression or using majority voting in classification, Random Forest reduces the overfitting common in individual decision trees, leading to more generalized predictions. The randomization process introduces decorrelation among the trees, which effectively reduces variance and enhances prediction stability. Additionally, each tree is trained on a bootstrapped dataset, and at each split, a random subset of features is considered. This ensures that each tree learns different patterns, contributing to the ensemble’s overall strength.

Random Forest is widely applied in various fields, such as finance, healthcare, and ecology, owing to its robustness and reliability in making predictions from large datasets. It excels in risk assessment, fraud detection, medical diagnoses, and ecological modeling. Despite its advantages, the model can be computationally expensive and resource-intensive due to the large number of trees. Its complexity also reduces interpretability, as it is difficult to visualize the decision-making process across all trees. Nonetheless, Random Forest offers a powerful balance between bias and variance, with an internal feature importance measure that helps identify key variables in predictive modeling tasks. (Sarica et al. 2017; Rigatti 2017; Feng, et al. 2020; Ao et al. 2019; Cha et al. 2021).

### Linear regression

It’s a foundational statistical approach that launches a linear relationship between a target variable and its influencing factors. In simple linear regression, a single independent variable forecasts the dependent variable, whereas multiple linear regression incorporates several predictors. The model assumes a linear relationship and is expressed as *Y* = *β*0 + *β*1*X*1 + *β*2*X*2 + … + *βnXn* + *ε*, where *β* represents coefficients and *ε* denotes the error term. The goal is to determine the coefficients that minimize the sum of squared residuals, a process achieved through least squares estimation. Model performance is assessed using metrics such as R-squared and residual analysis to evaluate goodness-of-fit and predictive accuracy.

Linear Regression is widely applied across disciplines due to its simplicity and interpretability. In economics, it models relationships between financial variables, while in marketing, it helps analyze consumer behavior. In biology, it is used for dose–response modeling, and in engineering, it predicts system outputs based on input data. Despite its advantages, Linear Regression assumes a strict linear relationship, making it unsuitable for complex, non-linear data. It is also sensitive to outliers and suffers from multicollinearity, which can distort coefficient estimates. To address these challenges, techniques such as variable transformation, regularization, and polynomial regression are often employed. In spite of its limitation, Linear Regression remains a fundamental manner for understanding data relationships and making initial predictive assessments (Guo and Wang 2019; Hope and Chapter 2020; Chen et al. 2019).

### Ridge regression

This approach enhances linear regression by incorporating an L2 regularization penalty to address multicollinearity and mitigate overfitting. By adding a penalty term proportional to the square of the coefficients’ amounts, it shrinks coefficients toward zero without eliminating them, promoting model stability. The penalty is controlled by a hyperparameter, lambda, which determines the degree of regularization and balances bias and variance. This adjustment makes Ridge Regression well-suited for datasets with correlated predictors by reducing the sensitivity of the fitted model to multicollinearity. Unlike ordinary least squares (OLS), Ridge favors smaller coefficients, improving stability and predictive performance when handling a large number of correlated features or medium-sized effects. Importantly, all predictors are retained, maintaining a more holistic model.

Ridge Regression is particularly valuable in high-dimensional datasets, such as genetic studies, where it identifies significant predictors like key genes while managing correlations effectively. In finance, it aids in credit risk modeling by stabilizing models with many variables, and in sales forecasting, it reduces overfitting while accounting for multicollinearity. Ridge offers a robust approach for prediction in complex datasets, though it does not perform feature selection, limiting its ability to produce sparse models. Its effectiveness depends on careful tuning of the regularization parameter, requiring computational resources and expertise for optimal implementation (Hoerl and Kennard 1970a, 1970b; Smith and Campbell 1980; Hoerl 2020).

### Lasso regression

This is a linear regression method that incorporates L1 regularization to develop both prediction exactness and model interpretability. The key feature of Lasso is its ability to perform variable selection by penalizing the absolute size of the regression coefficients, thereby driving some of them to exactly zero. This results in a sparse model that selects only the most significant predictors, making it valuable when working with data holding a large number of predictors. By shrinking less important variables’ coefficients to zero, Lasso mitigates overfitting, ensuring that irrelevant or redundant features do not influence the model. The strength of the regularization is controlled by a hyperparameter, lambda, which adjusts the trade-off between bias and variance, allowing for the retention of only relevant predictors.

Lasso Regression is widely applied across various domains, such as bioinformatics, where it helps identify significant genetic markers from a large set of genes, and econometrics, where it selects key economic predictors for forecasting trends. In engineering, particularly in signal processing, Lasso aids in feature selection while simplifying models. Its main advantage is its ability to produce a simpler, more understandable model by reducing the number of predictors. However, Lasso can be less effective when dealing with highly correlated variables, as it may arbitrarily choose one over others. The model’s performance heavily relies on the careful selection of the regularization parameter, which requires fine-tuning to achieve optimal results. In scenarios with high multicollinearity, Ridge Regression may sometimes outperform Lasso due to its ability to handle correlated features more effectively (Kang, et al. 2020; Roth 2004; Emmert-Streib and Dehmer 2019).

### Support vector regression

SVR extends SVM to regression tasks, predicting continuous values while maintaining errors within a specified margin ($\upepsilon$). It utilizes an insensitive loss function, reducing the impact of minor deviations and focusing on overall trends. By applying kernel functions like polynomial, linear, and radial basis functions (RBF), SVR effectively models both linear and nonlinear relations.

SVR is widely used in fields requiring precise forecasting, such as stock price prediction, real estate valuation, and energy consumption analysis. However, its computational cost is high, especially for large datasets, and performance depends on careful parameter tuning to balance accuracy and efficiency (Smola and Schölkopf 2004; Zhang and O’Donnell 2020; Kavitha et al. 2016).

### Gradient boosting machine

GBMs are cooperative learning models which combine multiple weak learners, archetypally decision trees, to build a strong predictive model. The core principle of GBMs is residual learning, where each new model corrects the errors of the previous one by focusing on the residual errors from earlier iterations. This process minimizes a differentiable loss function (e.g., mean squared error for regression or binary log loss for classification), progressively enhancing model accuracy. At each stage, GBMs add new models that adjust based on the previous iteration’s errors, creating a refined prediction with each step. The sequential learning process ensures that the model effectively captures complex data patterns and interactions.

GBMs have a broad range of applications, from web search rankings and credit scoring to fraud detection and healthcare analytics, offering high accuracy and predictive power. Their success in data science competitions highlights their ability to capture intricate data structures. However, their computational intensity, especially with large datasets, and the complexity of model interpretation pose challenges. Regularization and hyperparameter tuning are essential to prevent overfitting and ensure optimal performance, requiring significant expertise to balance model complexity and interpretability (Zulfiqar et al. 2021; Touzani et al. 2018; Fan et al. 2019).

#### K-nearest neighbors

KNN is a simple, non-parametric algorithm applied for regression uses. It operates by classifying a new data point based on the majority class of its ‘k’ closest training points, using distance metrics like Euclidean distance. KNN does not require explicit model training; instead, it stores all data points and performs predictions by calculating distances between the test point and each stored point. The choice of ‘k’ is crucial: a smaller ‘k’ makes the model sensitive to noise, while a larger ‘k’ may smooth out valuable details. KNN is widely used in applications such as recommendation systems, image recognition, anomaly detection, and medical diagnostics. Despite its simplicity and flexibility, KNN can be computationally expensive, especially with high-dimensional data, requiring efficient distance computations. Additionally, performance heavily depends on the correct selection of ‘k’ and distance metric (Holt et al. 1987; Bansal et al. 2022; Kramer and K-Nearest Neighbors, in Dimensionality Reduction with Unsupervised Nearest Neighbors. 2013).

#### Extreme gradient boosting

It is an advanced, scalable, and distributed gradient-boosting decision tree context designed to enhance the performance and speed of traditional gradient-boosting methods. By incorporating algorithmic optimizations like parallel tree boosting, it efficiently processes large datasets. Regularization terms, including L1 (Lasso) and L2 (Ridge), are integrated into the loss function to improve model performance and prevent overfitting. XGBoost’s architecture leverages decision trees as base learners, enhanced by efficient computational strategies such as weighted quantile sketch and column block compression. Its versatility makes it widely used in areas such as fraud detection, bioinformatics, and customer churn analysis, where non-linear relations and large-scale data require precise predictive capabilities. Although powerful, XGBoost’s complexity demands expertise for fine-tuning hyperparameters, as it can be challenging to interpret and optimize without careful parameter adjustments (Sado et al. 2023; Tyralis and Papacharalampous 2021).

#### Light gradient boosting machine

It’s a gradient-boosting system that utilizes decision trees as its core learning algorithm that are optimized for efficiency and speed across large datasets. Emphasizing lighter algorithmic infrastructure, it processes input data faster using advanced innovations like the histogram-based algorithm and leaf-wise growth strategy. LightGBM, designed to enhance computational speed without trading off accuracy, introduces exclusive feature bundling (EFB) and gradient-based one-side sampling, which reduce data features and observations during splitting—improving model precision while diminishing both time and space complexity during learning processes, suited for faster-distributed computing.

LightGBM’s key architectural innovations let it deploy faster, with efficient memory usage through customized data structures such as histograms, enabling faster data partitioning and decision tree expansion. By selecting the best leaf to grow instead of using the standard level-wise strategies, LightGBM achieves high precision in predicting with minimized complexity. The exclusive feature bundling reduces feature dimensionality in datasets, assisting in streamlined processing even with categorical data critical in real-world applications. Moreover, gradient-based one-side sampling confines observed samples to a compact representative, mitigating computational lag during model refinement and reducing overfitting potential.

LightGBM’s efficiency and capacity have made it a favorite choice in high-speed predictive modeling areas such as click-through rate predictions in advertising technology, customer churn prediction in telecommunications, and risk assessment in financial markets, where speed and precision are critical. It is extensively used in environments demanding resource scalability with fast-paced data analytics like retail demand forecasting and supply chain optimization. Its strength in running large-scale data predictions allows LightGBM to address real-world complexity challenges ranging from image processing tasks to genetic studies with massive sample size demands.

LightGBM’s principal advantage is its excellent performance speeds combined with scalable accuracy, particularly in high-dimensional data scenarios. Its capability to handle categorical variables differentiates it distinctly from similar boosting algorithms. However, precise tuning is required to avoid overfitting and optimize performance, necessitating knowledge of various hyperparameters. LightGBM models can be less interpretable than simpler algorithms due to the intricate nature of multi-layered processes, posing challenges when deployed in environments where model transparency is vital. Nonetheless, for applications prioritizing speed and extensive variable handling, LightGBM continues to be a preferred choice. (Fan et al. 2019; Guo et al. 2023; Taha and Malebary 2020).

#### Elastic net

It is a versatile regression approach integrating the regularization strengths of both Lasso (L1) and Ridge (L2) to improve model performance, especially where predictors outnumber observations or exhibit significant collinearity. Elastic Net balances between these penalties, facilitating the advantages of variable selection and shrinkage concurrently, without wholly assigning any input values zero coefficients, hence improving upon the limitations encountered with Lasso—specifically in retaining correlated feature pairs. The inclusion of both penalty types ensures dimensionality reduction power, governed by a dual-tuning parameter controlling the balance effect between Lasso-like sparse representations and Ridge-inspired continuous scaling.

Elastic Net is based on a linear model with a loss function penalized by both L1 and L2 norms. Through hyperparameters, lambda for regularization, and alpha determining the mix ratio between L1 and L2 penalties, Elastic Net fits models jointly using weighted combinations of penalties. This combined penalty optimizes performance by robustly shrinking coefficients, leading to better generalization of unseen data by controlling prediction variance. Elastic Net addresses issues like suppression of important predictors and multicollinearity through regularization effects, thus offering a flexible toolkit for hypothesis testing and exploratory modeling where typical regression methods might fail.

Due to its sophisticated handling of multicollinearity and feature selection, Elastic Net is extensively applied in machine learning tasks where the number of predictors is substantial. It is used in genomics to interpret vast gene expression data, ensuring model stability while highlighting predictive genes. It holds value in finance, too, where economic forecasting and asset price modeling demand predictive robustness from correlated market features. Elastic Net is instrumental in ensuring impressive, generalizable results, making it a pragmatic choice for complex systems requiring balanced precision, featured influence, and performance from data-rich in dimensional structure and interweaved variabilities.

Elastic Net’s combined penalty approach simultaneously empowers it with dimensionality reduction and predictive refinement capabilities, navigating multicollinear complications and ensuring stable prediction outputs. It enhances flexibility in dealing with diverse data structures, balancing sparsity and continuity in modeling approach useful when both feature selection and interpretability matter. However, setting the parameters for L1 and L2 combinations can be data-intensive, requiring expertise to operate efficiently without bias. The increased computational cost due to its dual regularization nature and potential complexity make Elastic Net less suited for very large datasets unless ample processing resources and expert analysis are involved (Qi and Yang 2022; Mokhtari et al. 2020).

#### Categorical boosting

It is a specialized gradient-boosting manner that is excellently engineered to manage categorical data, which is typically a challenge to standard gradient-based methods. CatBoost intrinsically incorporates sophisticated algorithmic insights to address categorical feature processing, applying a combination of ordered boosting—ensuring model stability by preventing overfitting—and innovative techniques for handling categorical data transformation during learning. Employing permutations and gradient-learning frameworks adeptly, CatBoost constructs a robust learning path, expertly optimizing prediction precision while maintaining a high resilience to typical overfit pitfalls induced within the data transformation processes known to plague standard boosting regimes.

The design of CatBoost features ordered boosting to reflect the temporal sequence of data rather than isolated instances, preserving underlying temporal patterns across data transformations. Its exclusive feature bundling method aggregates sparse categorical features, minimizing dimensionality while maintaining essential data fidelity. This mechanism enhances computational speed and prediction accuracy. CatBoost leverages sophisticated loss function computation enhancements across robust-level ensemble refinement, differentiating itself broadly from typical gradient boosting frameworks—catapulting precision in categorical transformation outcomes and boosting robustness toward permutation-induced variability for complex practical applications.

CatBoost excels in handling datasets rich with categorical variables and bias prevention needs, facilitating application in a variety of contexts, such as online advertising, where categorical information predominates in click-through predictions. The algorithm also supports customer segmentation tasks and recommendation system improvements across dynamic industries like e-commerce and digital marketing. In domains requiring rigorous analysis and robust prediction across diverse data types, like insurance and healthcare analytics, CatBoost ensures precision in identifying and learning from categorical variable interactions essential to robust decision-making architectures.

CatBoost’s core strengths lie in its tailored approach to managing categorical variables, effectively preventing overfitting through ordered boosted solutions and precise feature handling. Its adeptness in maintaining high accuracy across complex categorical data domains distinguishes it in market competition layers, offering seamless applications with substantially reduced human intervention necessary for categorical encoding. The challenges with CatBoost center primarily on computational resources cost, it demands ample resources when operated on very extensive datasets. Effective initial setup and parameter tuning are crucial, requiring domain expertise to optimize the model engaging in outcomes better suited for high-value, real-world categorical data deployment tasks (Cha et al. 2021).

## Scalability and energy efficiency of hydrochar production

The transition of electrocatalytic reactors from laboratory research to industrial application presents a number of significant engineering challenges. These barriers are not related to the catalyst material itself, but rather to the design of the reactor, including limitations in mass transport, energy losses, and the long-term stability of components.

A primary challenge is managing mass transport, especially for reactions involving gaseous reactants like CO_2_. The low solubility and slow diffusion of these gases in liquid electrolytes can severely limit the reaction rate at high current densities. To overcome this, engineers have developed Gas Diffusion Electrodes (GDEs), which provide a critical interface where gas, liquid, and solid phases meet, allowing for efficient delivery of reactants to the catalyst.

Another major set of barriers involves energy efficiency. There are two main types of energy losses to address. Ohmic losses are caused by electrical resistance in the reactor’s components, which can be minimized by optimizing electrode spacing and electrolyte conductivity. Kinetic overpotential, on the other hand, is the inherent energy barrier of the reaction itself. This is primarily an issue of catalyst design, and can be improved by developing more active and efficient catalytic materials.

Finally, ensuring the long-term stability and durability of the reactor is a key engineering hurdle. GDEs are particularly vulnerable to degradation. Common failure modes include “flooding,” where the pores of the electrode become saturated with electrolyte, blocking gas flow. This is often caused by the degradation of the hydrophobic materials within the GDE. Additionally, carbon-based GDEs can suffer from corrosion in the harsh electrochemical environment, leading to a loss of structural integrity. To address these issues, research is focused on developing more durable, and even carbon-free, GDEs.

## Scalability and energy efficiency of hydrochar production

The practical applicability and commercial viability of hydrochar production via hydrothermal carbonization (HTC) are contingent on the process’s scalability and energy efficiency, aspects often overlooked in laboratory-scale studies. The HTC process offers a distinct advantage over other thermochemical methods, such as pyrolysis, by its ability to process wet biomass feedstocks without an energy-intensive pre-drying step. This inherent efficiency is a key driver for the technology’s potential for industrial-scale deployment.

The HTC reaction itself is exothermic, releasing energy that can be captured and recycled to heat incoming feedstock, thereby reducing overall energy consumption. In a large-scale implementation with sewage sludge, it was shown that only about 20% of the fuel energy content of the final hydrochar product was required to power the process. Specific energy consumption figures from a study on grape marc showed thermal energy and power consumption of 1170 kWh and 160 kWh, respectively, per ton of hydrochar produced. When compared to conventional methods for processing wet waste, HTC can reduce energy consumption for drying by up to 70% by leveraging heat recirculation.

The transition of HTC from laboratory to commercial scale, while challenging, is well underway. Key challenges include optimizing reactor design, managing the process water, and ensuring consistent product quality. Despite these obstacles, numerous companies have successfully developed and implemented industrial-scale HTC plants, validating the technology’s scalability. The process is considered an effective solution for converting a wide range of biomass into valuable materials due to its relatively mild process conditions and industrial scalability.

From an economic perspective, cost–benefit analyses demonstrate the potential for hydrochar to be a profitable product. One techno-economic assessment found a breakeven selling price of $117 per ton for co-hydrochar, while another study calculated a production cost of 157 €/ton with a break-even point of 200 €/ton for pelletized hydrochar. The specific mechanical energy required for the process has been reported to be in the range of 83.5 to 152.3 kWh/t, directly addressing the importance of energy cost metrics. Furthermore, one analysis found the total energy consumption per ton of hydrochar-derived sand to be 695.14 kWh, with an energy cost of approximately $41.70 per ton, demonstrating the potential for significant energy costs in downstream processing.

This integration of process efficiency and economic feasibility is critical for the long-term adoption of hydrochar as a sustainable product. By converting low-value, high-moisture biomass into a stable, energy-dense solid fuel, HTC offers a solution that not only mitigates waste disposal costs but also contributes to a circular economy by creating a valuable product with a positive energy balance.

## Data investigation and clarification

The data required to build the models were earlier compiled in Shafizadeh et al. (2023). In this research, the independent inputs include reaction processing circumstances and biomass characteristics. Biomass features are categorized into proximate and ultimate analyses, with the proximate investigation covering fixed carbon, volatile matter, and ash content (all in wt%). For this work, the proximate features are selected as input. As such, the input features are volatile matter, fixed carbon, water content, reaction-time, temperature, and ash-content. The outputs are hydrochar- higher-heating-value (HHV, in MJ/kg) and hydrochar yield (in wt%). The data-bank consists of 481 rows.

**Fig. 2:**
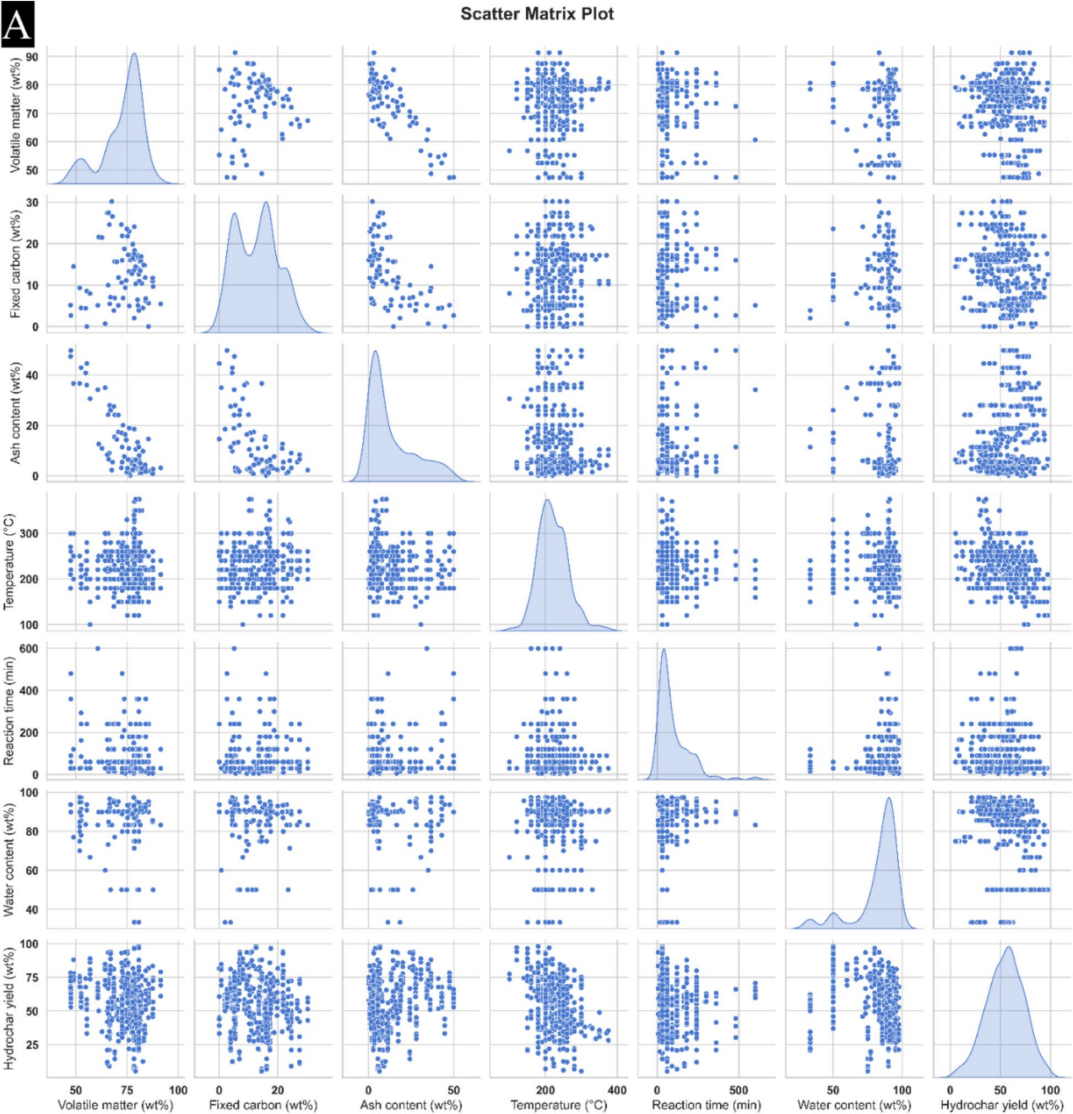

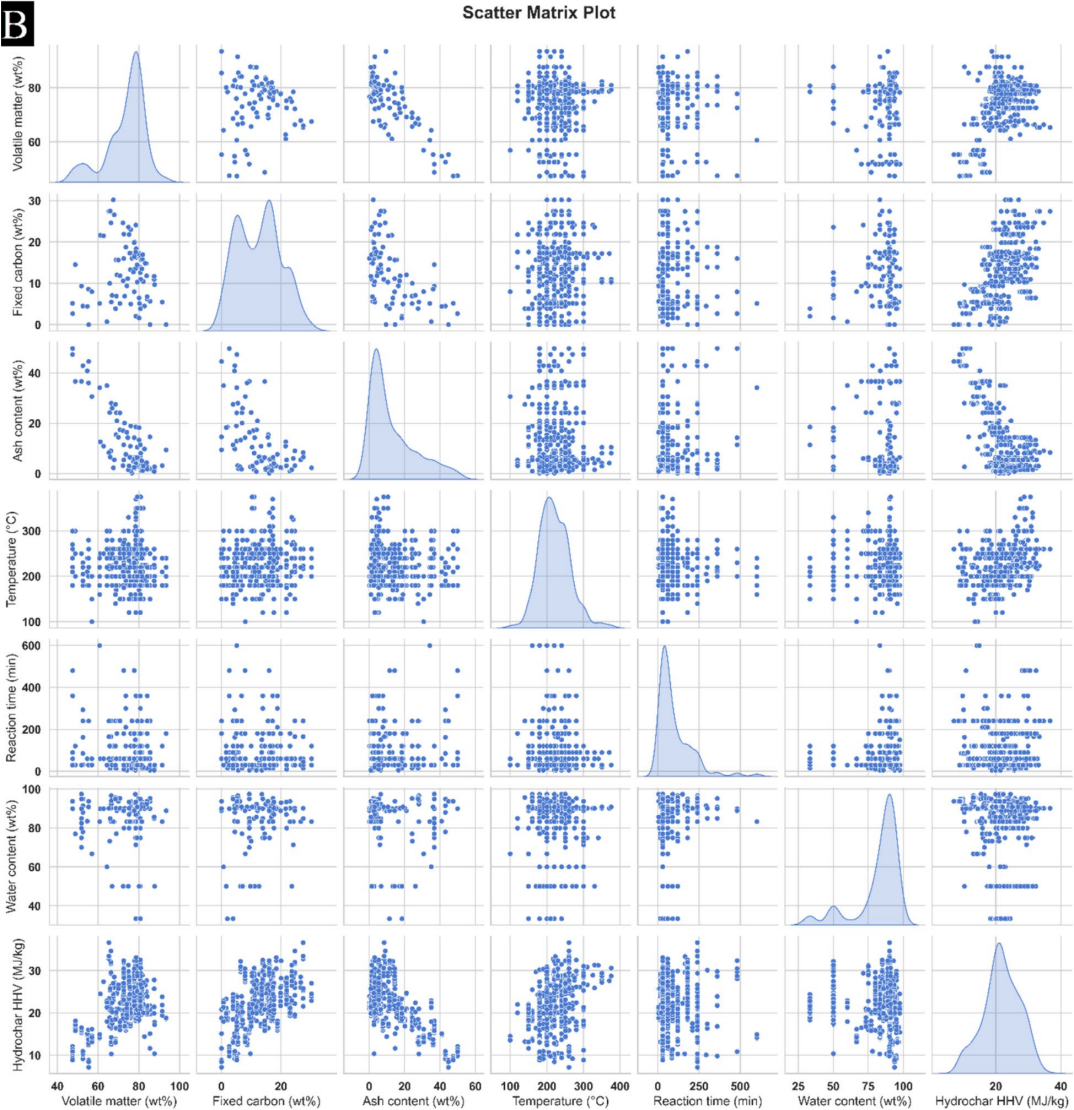
Matrix-plot for **A** yield **B** HHV

Figure 2A, B present the boxplots of all parameters reflected in the modelling of HHV and yield. In current study, a total of 337 data points were utilized for model training, while 72 points were assigned to both testing and validation processes.

**Fig. 3:**
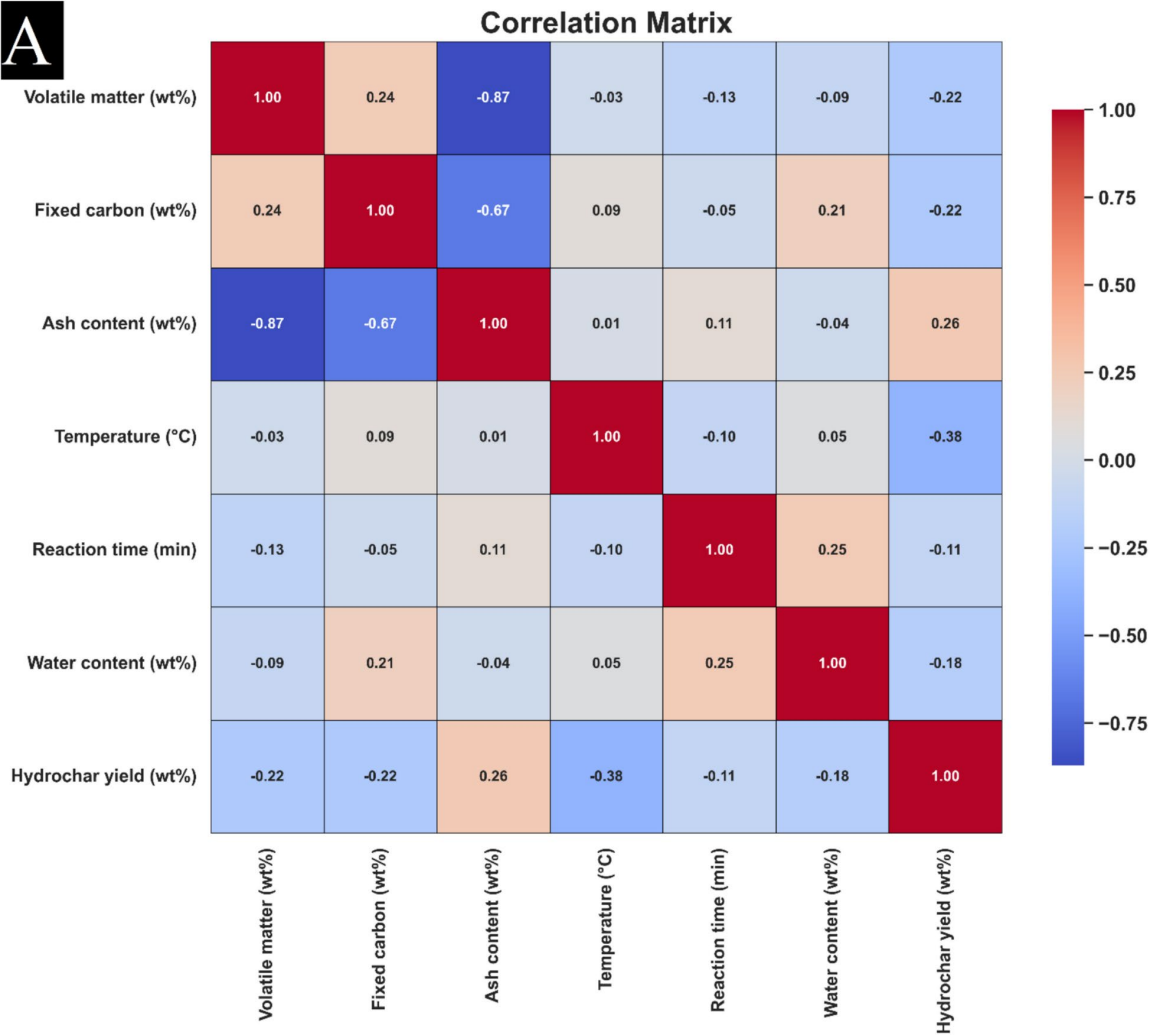

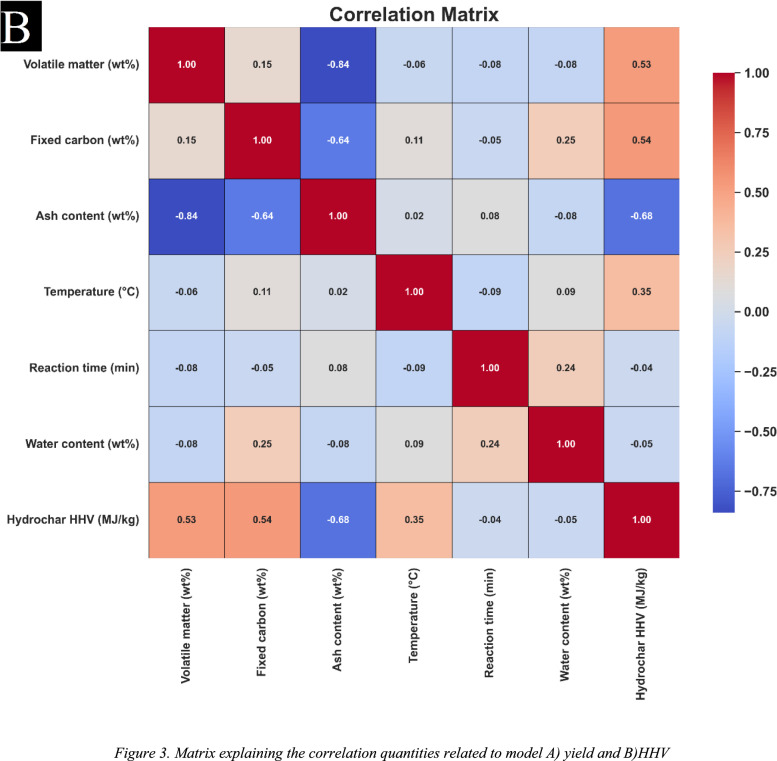
Matrix explaining the correlation quantities related to model **A** yield and **B** HHV

Figure 3 illustrates the Pearson correlation coefficients analyzed in this study. The Pearson coefficient is a statistical measure used to quantify the power and direction of the linear relationship between two parameters. This coefficient ranges from − 1 to + 1, where + 1 indicates a perfect positive linear correlation, − 1 represents a perfect negative linear correlation, and 0 signifies the absence of any relation. The following eaution describes the calculating of the Pearson correlation (Abbasi et al. 2023; Bemani et al. 2023; Madani and Alipour 2022; Madani et al. 2021):

1 $${r}_{j}=\frac{\sum_{i=1}^{n}({I}_{i.j}-\overline{{I }_{j}})({Z}_{i}-\overline{Z })}{\sqrt{\sum_{i=1}^{n}{({I}_{i.j}-\overline{{I }_{j}})}^{2}\sum_{i=1}^{n}{({Z}_{i}-\overline{Z })}^{2}}}$$

In this formula, Z represents the second variable and is characterized by its averaged amount, Z̄, in which Ī denotes the average amount of the approximate Ij. The relationships among the inputs and the target parameters namely, HHV and yield—are particularly noteworthy. As shown in Fig. 3A, it is evident that each input variable exhibits some degree of correlation with hydrochar yield, with the exception of Ash content, that shows a positive correlation. Other inputs demonstrate a negative correlation with hydrochar yield. Similarly, Fig. 3B highlights the correlations among hydrochar HHV and input variables. In this case, parameters such as Temperature, Fixed Carbon, and Volatile Matter exhibit a positive correlation with HHV, while the remaining factors present a negative correlation.

Before developing models based on data, it is crucial to guarantee the data quality by identifying and tackling potential outliers. In this work, the Monte Carlo Outlier Detection (MCOD) was utilized due to its success and efficiency when dealing with large data. MCOD syndicates random sampling with density based manners to detect anomalies, defining outliers as data points that deviate significantly from their neighbors in terms of local density. These anomalies are identified by examining the distribution of data points within their local neighborhood.

The MCOD algorithm leverages Monte Carlo sampling to select a representative subset of the data, thereby reducing the computational burden significantly. Its primary advantage lies in its efficiency, mostly for high-dimension datasets. By means of random sampling, MCOD evades the need to examine the full dataset directly, resulting in lower computational costs and making it especially appropriate for real-time applications. However, there are trade-offs; the accuracy of the results may depend on factors such as the number of samples and the arrangement of the adjacent neighbors (k). Nevertheless, MCOD remains an invaluable tool for data investigation and outlier detection, principally in scenarios where estimated results are acceptable or computational resources are limited. Its balance between computational effectiveness and detection accuracy is a key asset in identifying outliers in complex data-sets.

**Fig. 4:**
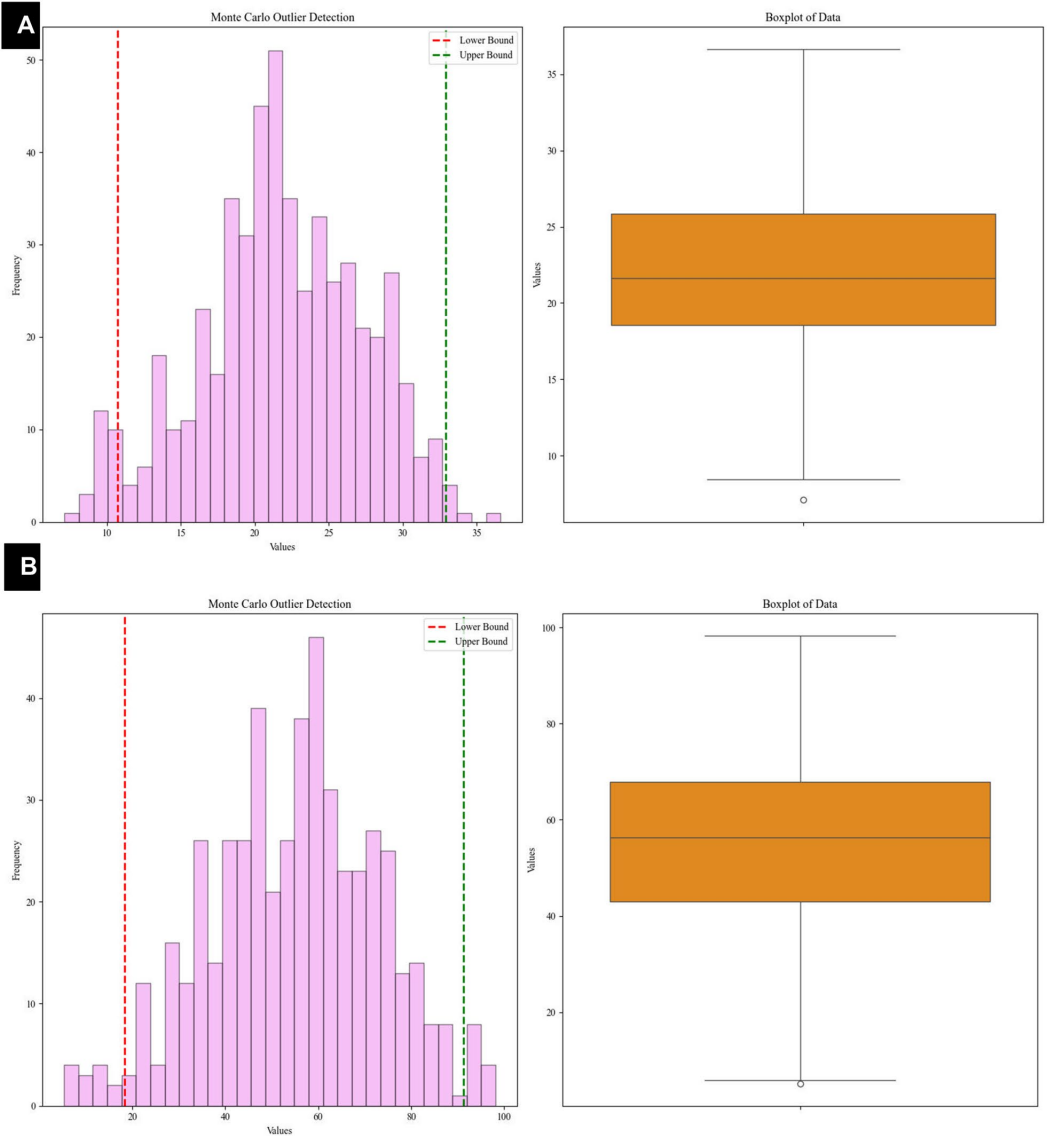
Outlier detection via the Monte Carlo and boxplots for the **A** yield and **B** HHV data validated the data’s spreading and confirmed its appropriateness for building dependable models

Figure 4A, B present boxplots of the data used for yield and HHV modeling, showcasing the spreading of points and highlighting the ranges suitable for model development. The boxplots indicate that the majority of points fall within the expected range, affirming the consistency and superiority of the data. For training, the entire dataset was utilized to ensure robust model developing. By incorporating the full range of data, the models are equipped to capture essential patterns and variability, enhancing their ability to oversimplify effectively to unseen data and improving calculation accuracy (Jia et al. 2018; Rocco and Moreno 2002).

## Results and discussions

This research uses diverse methods, from linear regression to convolutional neural networks and collective techniques (LightGBM, XGBoost, CatBoost), to forecast hydrochar higher heating value (HHV) and yield from biomass. This approach allows for a inclusive evaluation of model performance. Model accuracy and explanatory power are assessed using the mean squared error (MSE), coefficient of determination (R^2^), and mean relative deviation percent (MRD%). R^2^ measures the model’s capacity to clarify data variance, while MSE quantifies prediction accuracy, providing a benchmark for comparing models across datasets. The research includes detailed calculations and interpretations of the metrics to clarify implications for HHV and yield prediction (Feller et al. 2004; Aghdam et al. 2022; Bassir and Madani 2019; Shoushtari et al. 2020).

2 $$ R{ - }squared\;\left( {R^{2} } \right) = 1 - \frac{{\mathop \sum \nolimits_{i = 1}^{N} \left( {{\text{y}}_{{\text{i}}}^{{{\text{real}}}} - {\text{y}}_{{\text{i}}}^{{{\text{predicted}}}} } \right)^{2} }}{{\mathop \sum \nolimits_{i = 1}^{N} \left( {{\text{y}}_{{\text{i}}}^{{{\text{real}}}} - \overline{{{\text{y}}^{{{\text{real}}}} }} } \right)^{2} }} $$

3 $$ Mean\;squared\;error\;\left( {MSE} \right) = \frac{1}{N}\mathop \sum \limits_{i = 1}^{N} \left( {y_{i}^{real} - y_{i}^{predicted} } \right)^{2} $$

4 $$ Standard\;Deviation\;\left( \sigma \right) = \sqrt {\frac{1}{N}\mathop \sum \limits_{i = 1}^{N} \left( {{\text{y}}_{{\text{i}}}^{{{\text{real}}}} - \overline{{{\text{y}}^{{{\text{real}}}} }} } \right)^{2} } $$

5 $$ Mean\;relative\;deviation\;\left( {MRE} \right) = \frac{100}{N}\mathop \sum \limits_{i = 1}^{N} \left( {\frac{{y_{i}^{real} - y_{i}^{predicted} }}{{y_{i}^{real} }}} \right) $$

where, ${{\varvec{y}}}_{{\varvec{i}}}^{{\varvec{r}}{\varvec{e}}{\varvec{a}}{\varvec{l}}}$ and ${{\varvec{y}}}_{{\varvec{i}}}^{{\varvec{p}}{\varvec{r}}{\varvec{e}}{\varvec{d}}{\varvec{i}}{\varvec{c}}{\varvec{t}}{\varvec{e}}{\varvec{d}}}$ indicating the real and model output, respectively. Also, N signifies the dataset’s size.

A comparative investigation of several regression models was executed to evaluate their predictive performance on a benchmark dataset. The detailed results, summarized in Table 1 (for biomass yield prediction) and Table 2 (for biomass HHV prediction), highlight CatBoost as the best model for both yield and HHV prediction tasks. CatBoost demonstrated a strong correlation with the actual values, along with minimal standard deviation, indicating its high stability and accuracy.

**Table 1:** Created specific calculation metrics to appraise the efficiency of each technique on yield prediction

Model	Train R^2^	Validation R^2^	Test R^2^	Train MSE	Validation MSE	Test MSE	Train MRD%	Validation MRD%	Test MRD%
ANN	0.90	0.88	0.89	24.9	33.4	34.1	7.5	8.3	9.4
CNN	0.92	0.93	0.89	18.2	20.6	31.5	6.6	7.0	9.0
Linear Regression	0.24	0.36	0.13	183.9	184.8	258.6	23.8	24.5	30.6
Ridge Regression	0.24	0.34	0.14	184.9	190.1	254.7	23.9	25.0	30.7
Lasso Regression	0.24	0.36	0.13	184.1	185.4	257.3	23.8	24.6	30.5
Elastic Net	0.23	0.34	0.14	185.4	192.2	254.4	23.9	25.2	30.7
SVR	0.39	0.42	0.28	148.8	166.9	213.2	21.3	23.5	27.8
Random Forest	0.85	0.84	0.83	35.9	45.4	51.7	7.6	8.5	9.0
Gradient Boosting	0.77	0.74	0.77	56.5	76.5	68.8	11.2	12.5	12.9
KNN	0.71	0.69	0.72	70.9	91.1	81.7	13.5	14.0	15.6
Decision Tree	0.66	0.71	0.73	82.1	83.4	79.9	7.3	8.6	7.5
XGBoost	0.81	0.85	0.82	46.3	42.3	53.6	8.5	10.4	10.1
LightGBM	0.89	0.89	0.84	25.9	33.0	46.5	8.1	8.5	10.6
CatBoost	0.91	0.89	0.90	22.2	32.6	30.4	6.9	7.4	8.1

**Table 2:** Created specific calculation metrics to review the effectiveness of each technique

Model	Train R^2^	Validation R^2^	Test R^2^	Train MSE	Validation MSE	Test MSE	Train MRD%	Validation MRD%	Test MRD%
ANN	0.85	0.88	0.82	3.6	2.2	4.5	6.0	5.2	7.1
CNN	0.92	0.93	0.89	1.9	1.4	2.8	4.6	4.3	5.4
Linear Regression	0.57	0.67	0.58	10.4	6.3	10.5	11.0	9.5	11.9
Ridge Regression	0.57	0.67	0.58	10.4	6.3	10.5	11.1	9.5	11.9
Lasso Regression	0.57	0.67	0.58	10.4	6.3	10.5	11.0	9.5	11.8
Elastic Net	0.57	0.67	0.58	10.4	6.3	10.5	11.1	9.5	11.8
SVR	0.65	0.80	0.63	8.5	3.8	9.2	9.1	6.2	9.7
Random Forest	0.93	0.97	0.79	1.7	0.6	5.2	2.9	2.5	4.2
Gradient Boosting	0.90	0.90	0.84	2.5	2.0	3.9	5.0	5.1	5.8
KNN	0.82	0.89	0.82	4.5	2.1	4.6	6.4	5.1	7.1
Decision Tree	0.91	0.99	0.70	2.2	0.2	7.5	1.2	0.3	3.0
XGBoost	0.86	0.91	0.79	3.3	1.8	5.2	4.9	4.4	5.3
LightGBM	0.94	0.95	0.90	1.5	1.1	2.5	3.8	3.8	4.2
CatBoost	0.96	0.97	0.93	0.9	0.5	1.6	2.9	2.5	3.4
ANN	0.85	0.88	0.82	3.6	2.2	4.5	6.0	5.2	7.1

Specifically, Table 1 shows that CatBoost achieved an R^2^ value of 0.90 for biomass yield prediction, while Table 2 indicates an R^2^ value of 0.93 for HHV prediction. These values suggest that CatBoost effectively captures the basic relations in the data. It also exhibited the lowest MSE values, 30.38 for yield prediction and 1.63 for HHV prediction. Additionally, the Mean Relative Deviation (MRD) values were minimal, with 8.09% for yield prediction and 3.4% for HHV prediction, further confirming its superior performance in both regression tasks.

Conversely, simpler models including Lasso Regression, Linear Regression, Ridge Regression, Decision Trees, Elastic Net, and K-Nearest Neighbors (KNN) showed significantly poorer performance. These models showed lower R^2^ values and higher MSE values for both yield and HHV predictions. This performance disparity is consistent with prior research, which has demonstrated that gradient-boosting methods like CatBoost tend to outperform traditional models in complex regression tasks. The findings in this study emphasize the advantages of using advanced models like CatBoost for regression tasks in biomass yield and HHV prediction, especially when compared to simpler, more traditional approaches (Ajin et al. 2024; Vishwakarma et al. 2024).

Current work hires graphical techniques, such as cross plots and relative deviation values, to evaluate the reliability of various algorithms in predicting hydrochar HHV and yield biomass proximate analysis. These visual tools are instrumental in providing a comprehensive understanding of the models’ performances, effectively highlighting patterns and discrepancies between the predicted and real data.

**Fig. 5:**
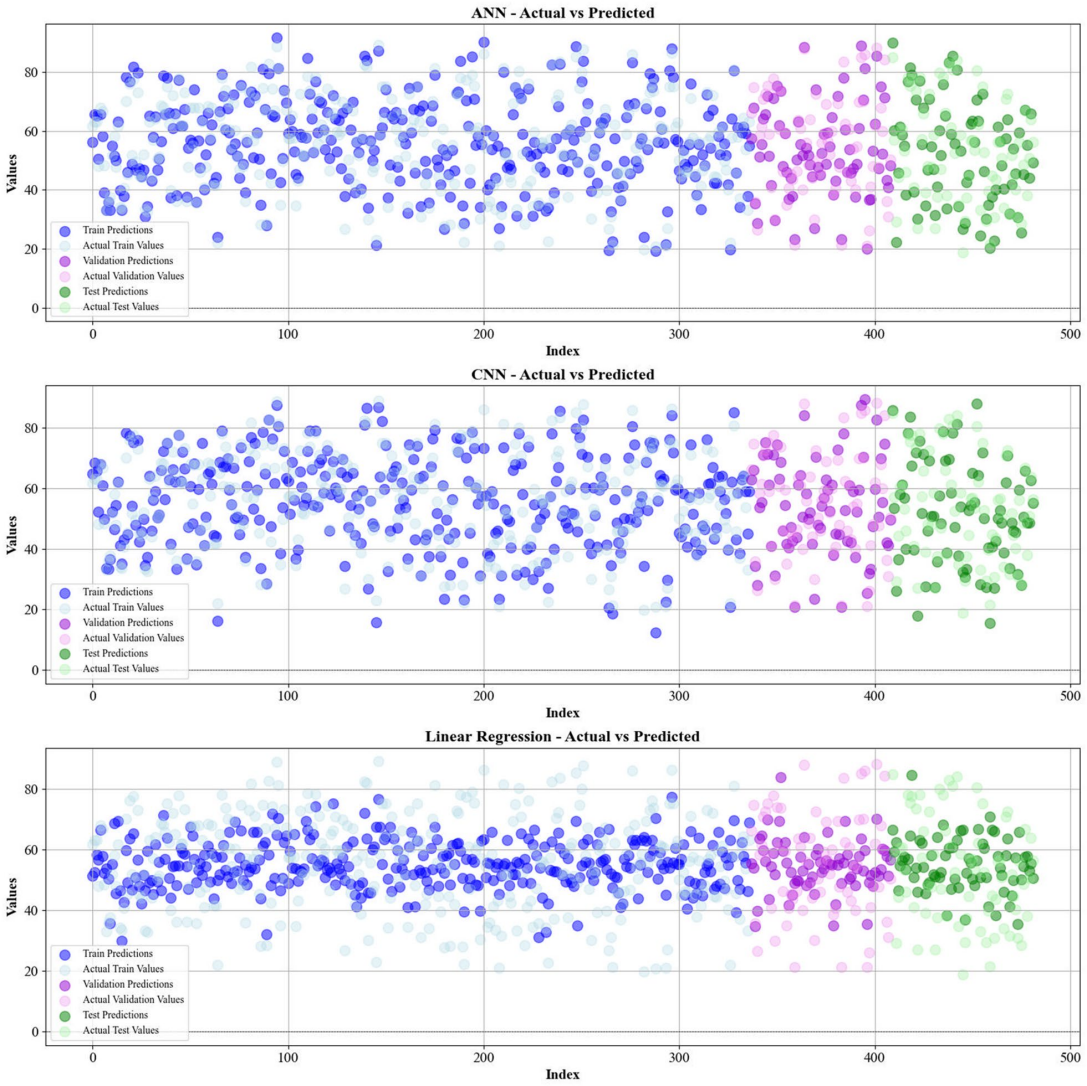

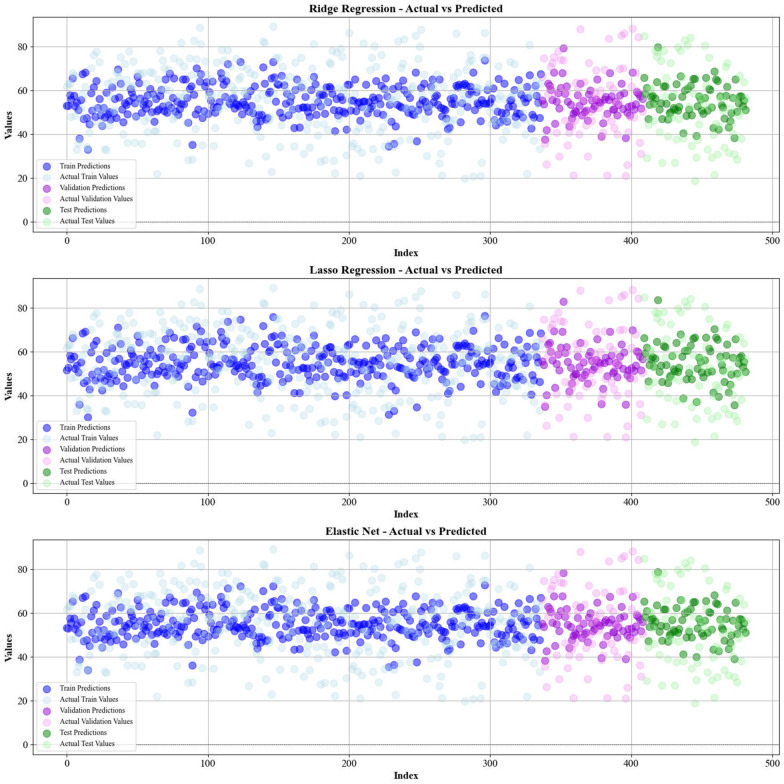

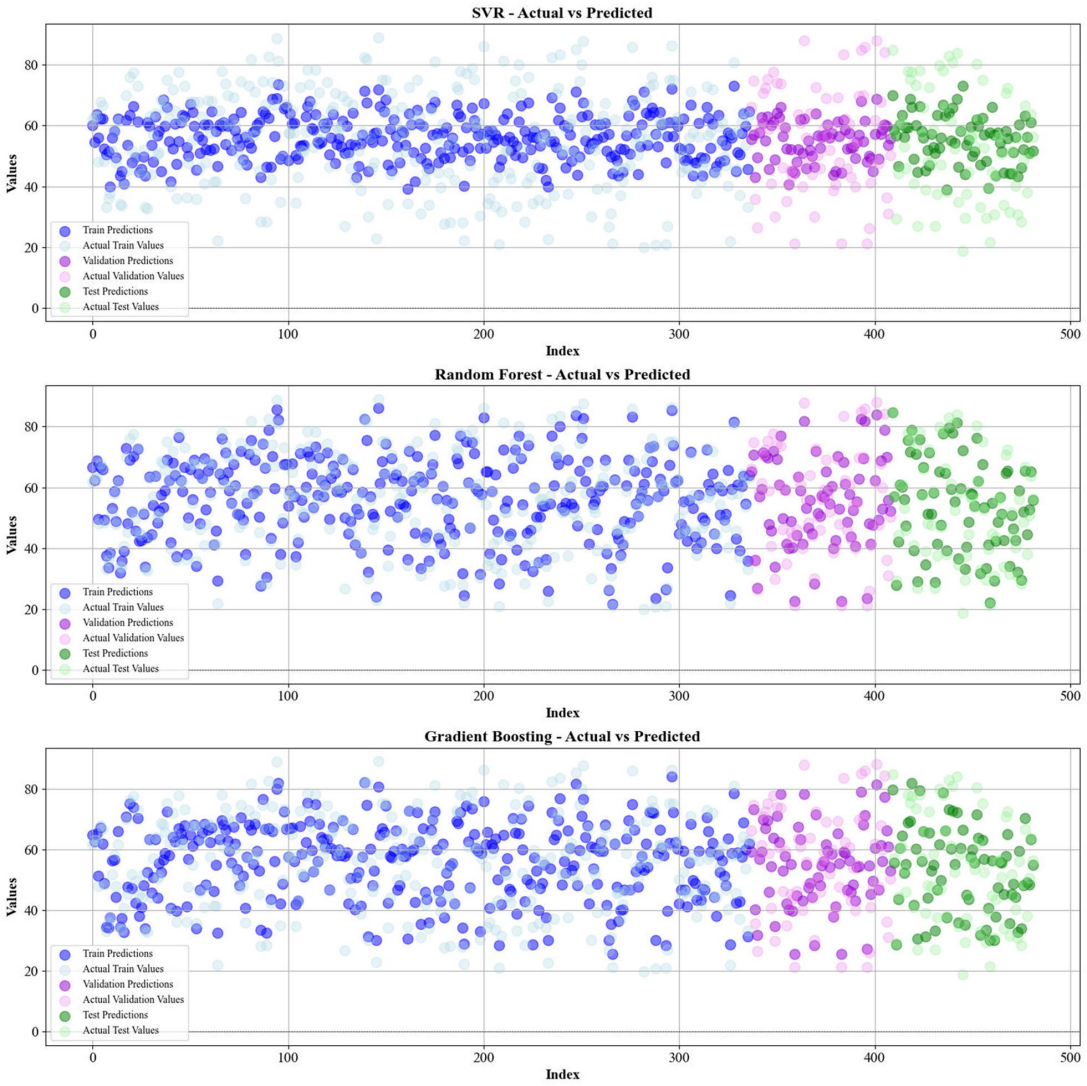

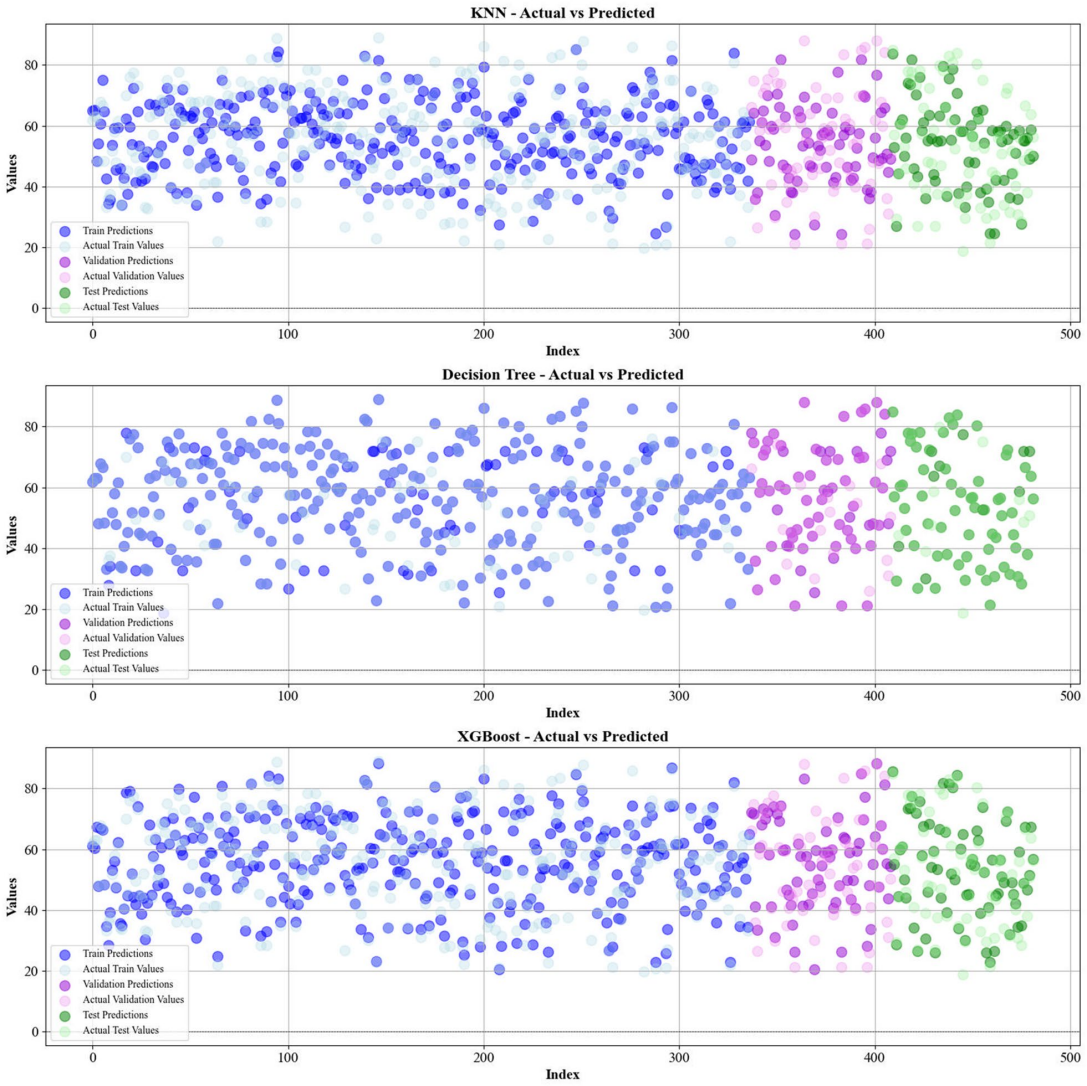

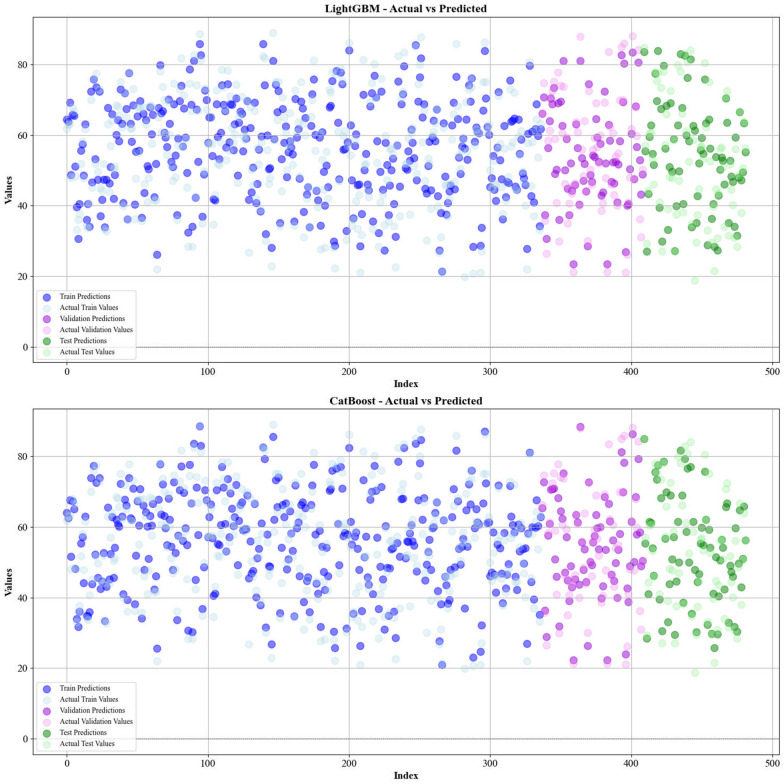
The accuracy of models for HHV prediction determined by comparing their predicted outcomes against observed values

**Fig. 6:**
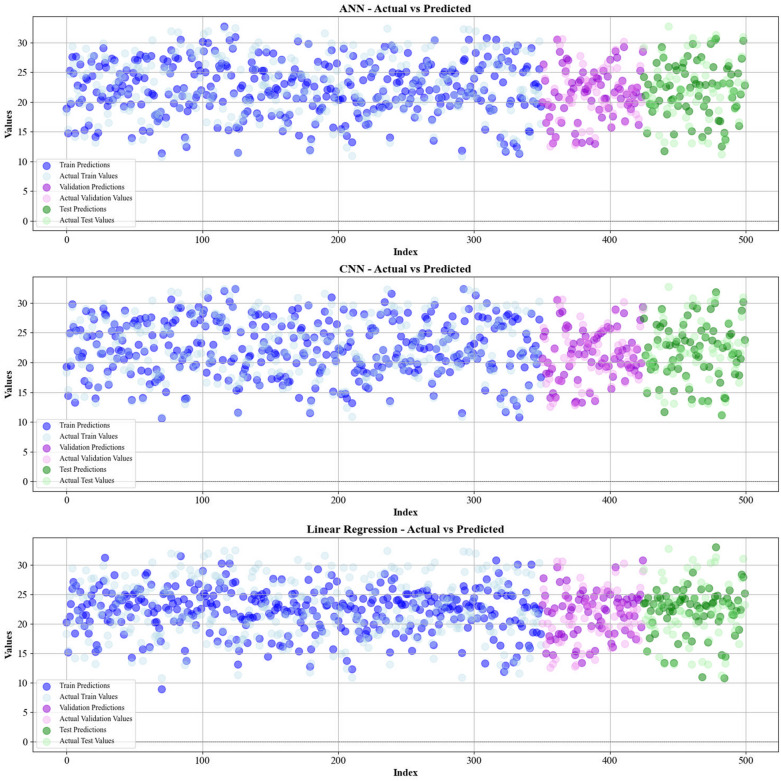

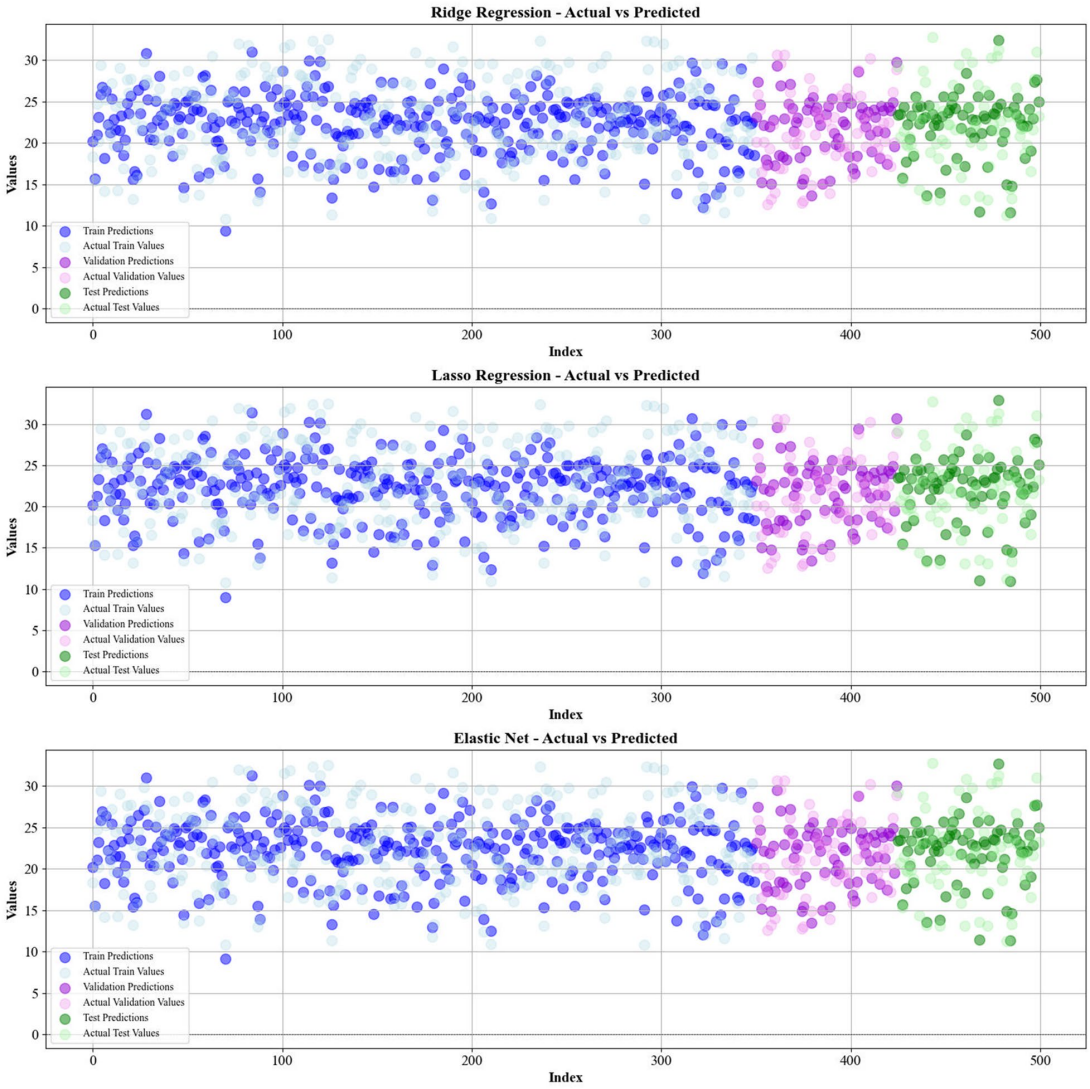

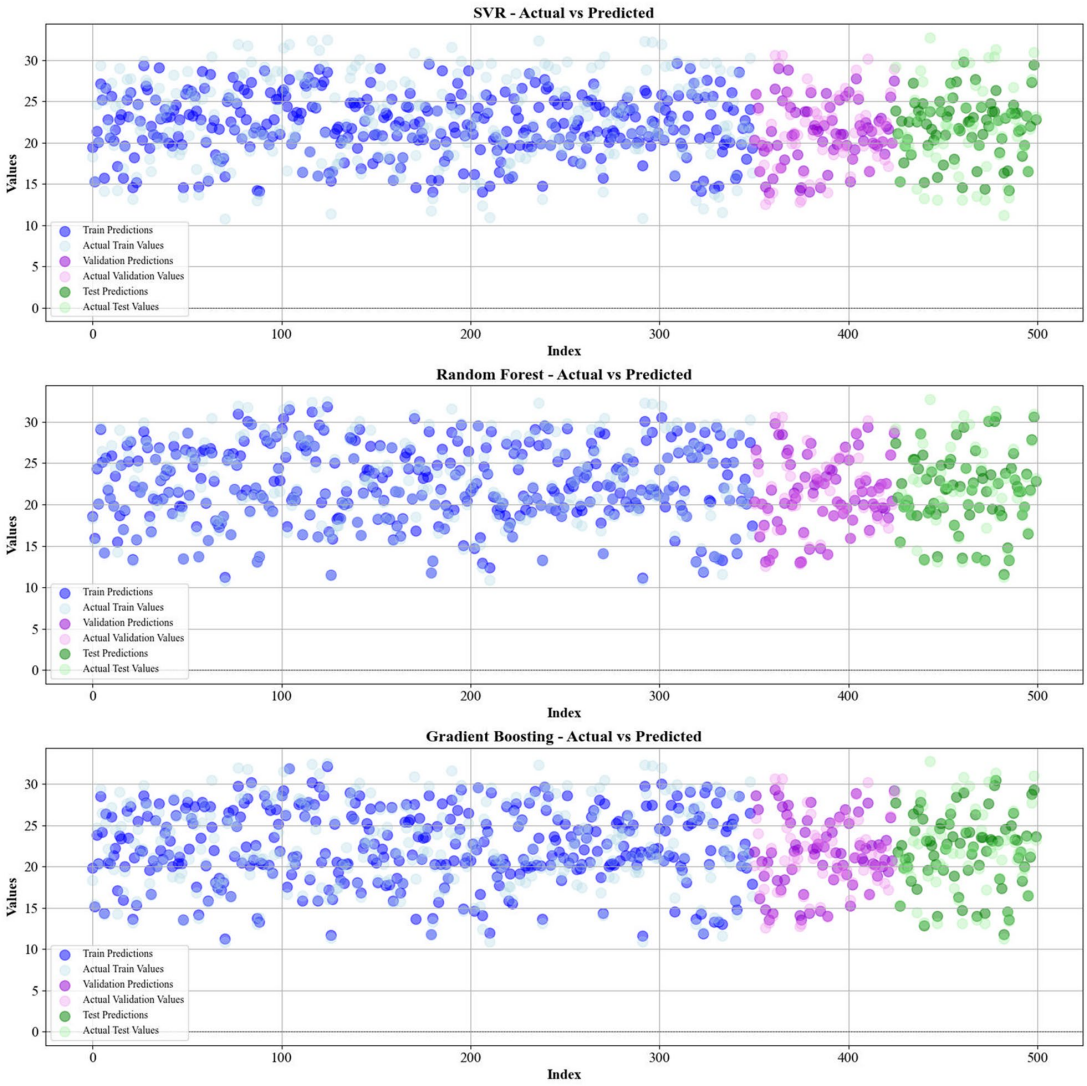

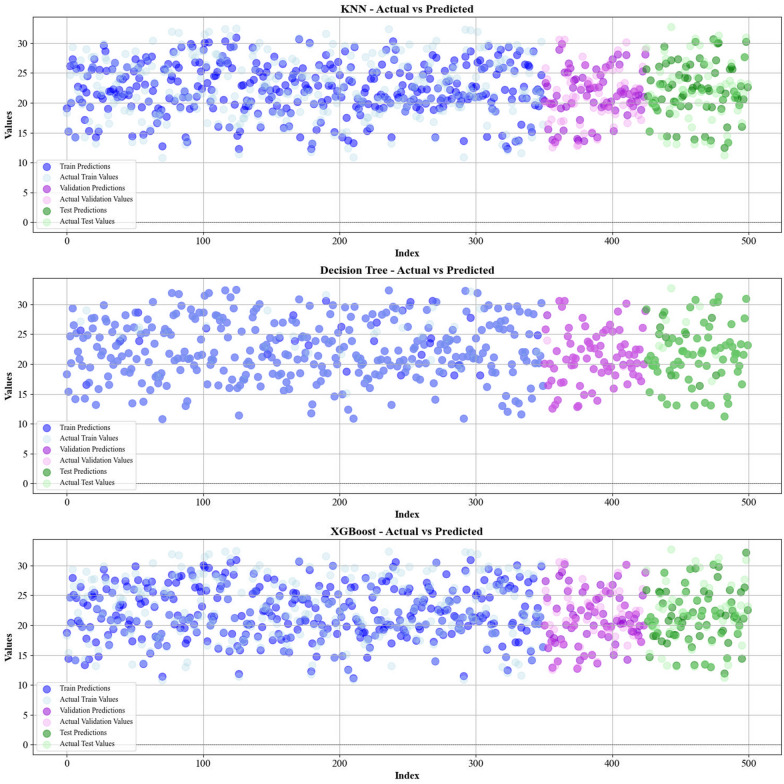

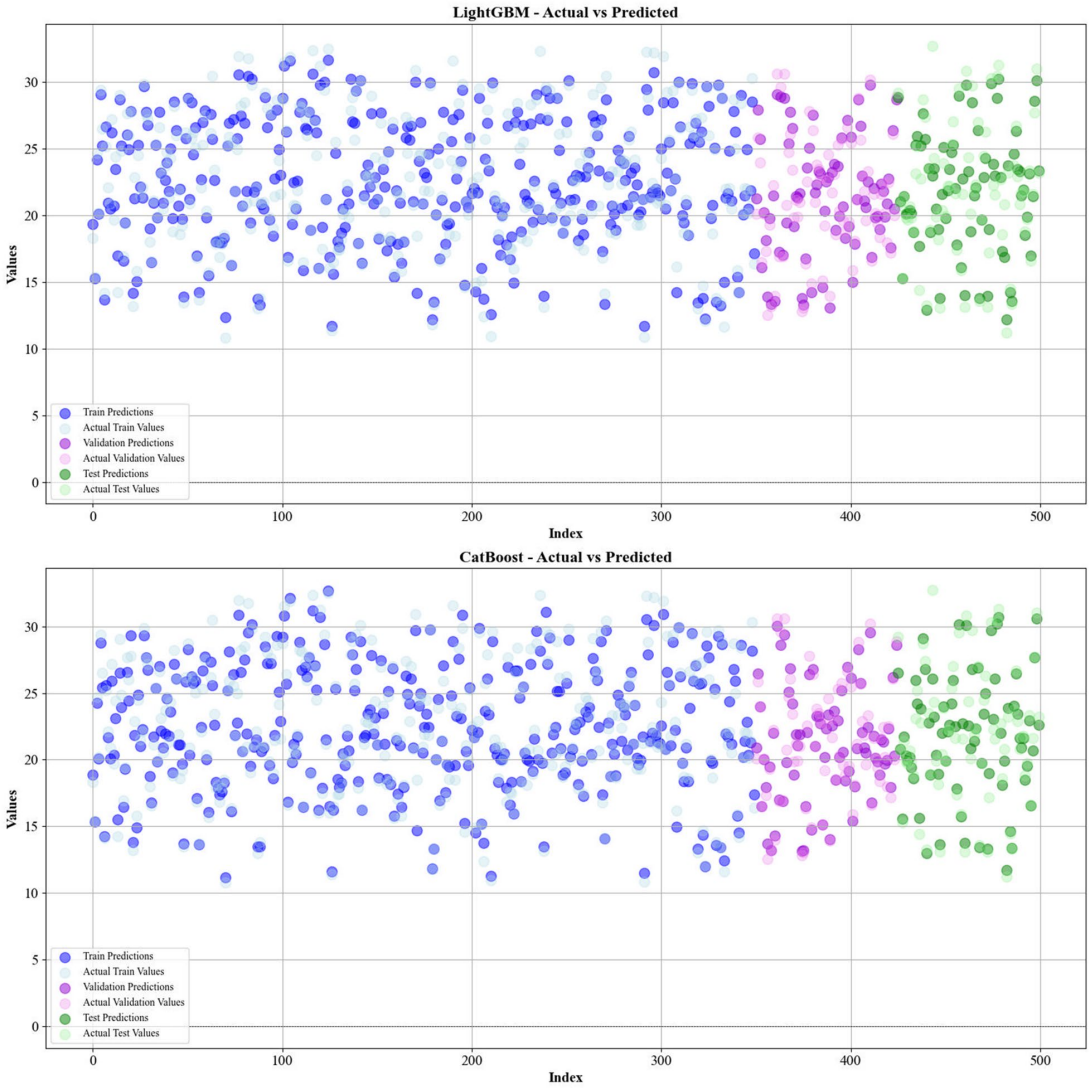
The accuracy of models for HHV prediction determined by comparing their predicted outcomes against observed values

Figures 5 and 6 present a comparison of the calculated and real data across different datasets, for all established models in predicting yield and HHV. The investigation reveals that the CatBoost shows near-perfect alignment between the real and modeled reults, demonstrating its great abilities. This close agreement underscores CatBoost’s strength in capturing the complicated relations within the data.

**Fig. 7:**
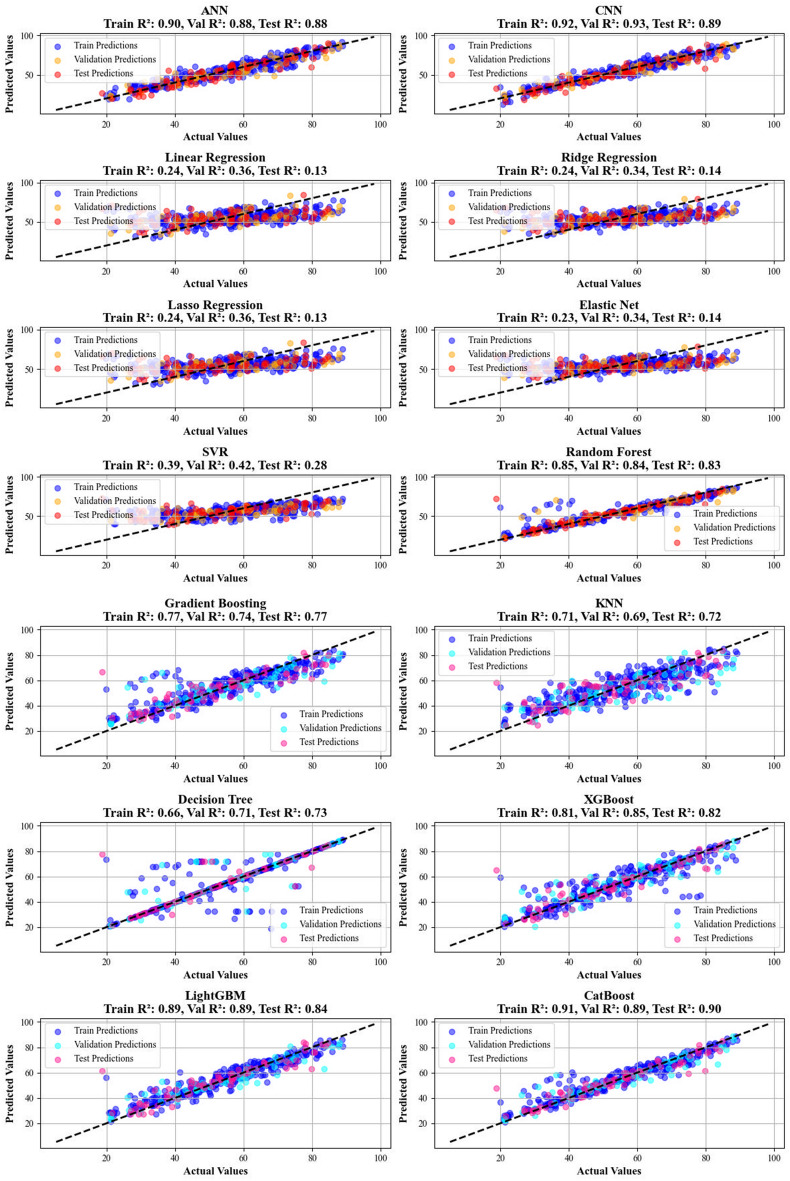
A visual assessment of the model’s predictive capability for yield, performed using cross-plots

**Fig. 8:**
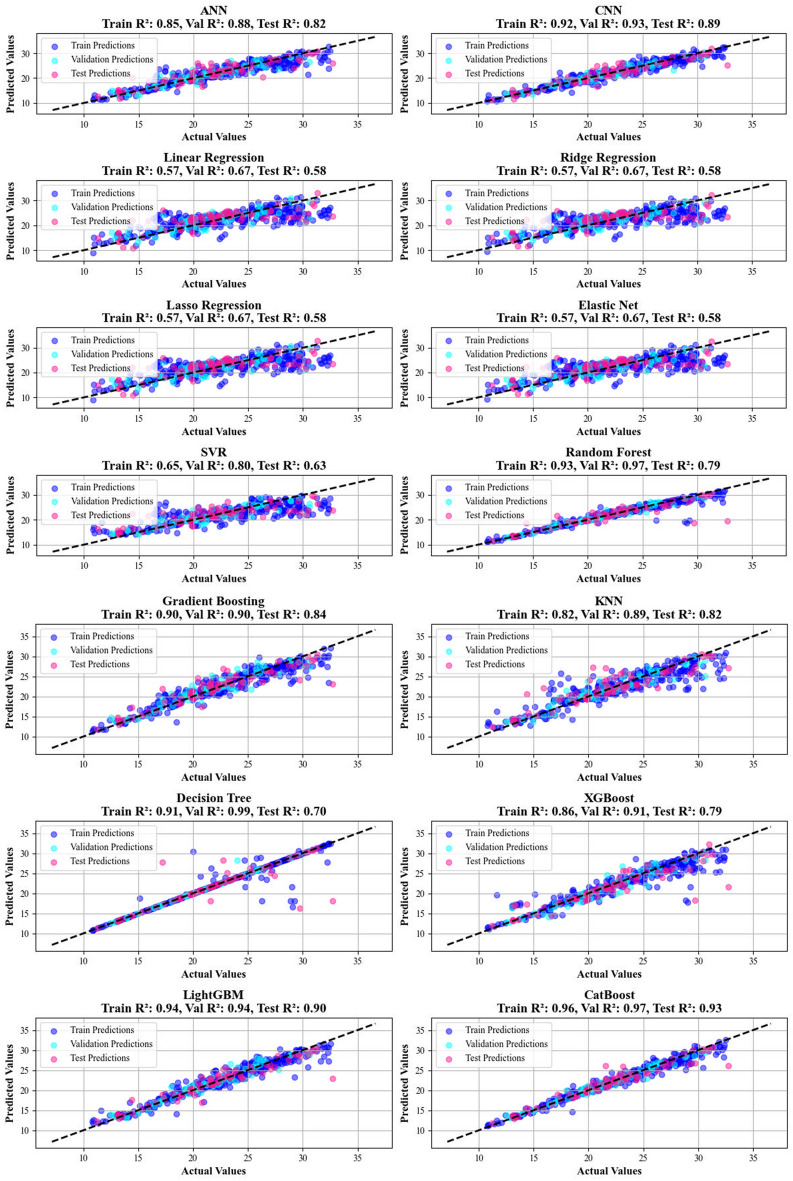
A visual assessment of the model’s predictive capability for HHV performed using cross-plots

Additionally, Figs. 7 and 8 introduce cross-validation plots that display the correlations between real and calculated amounts for all models in predicting hydrochar yield and HHV. The CatBoost shows a clear trend where data points for both parameters cluster tightly around the y = x bisector, with the fitted lines strictly mirroring this ideal relationship.This alignment serves as a testament to the model’s strong prediction, confirming its proficiency in accurately forecasting outcomes that align with real-world observations.

**Fig. 9:**
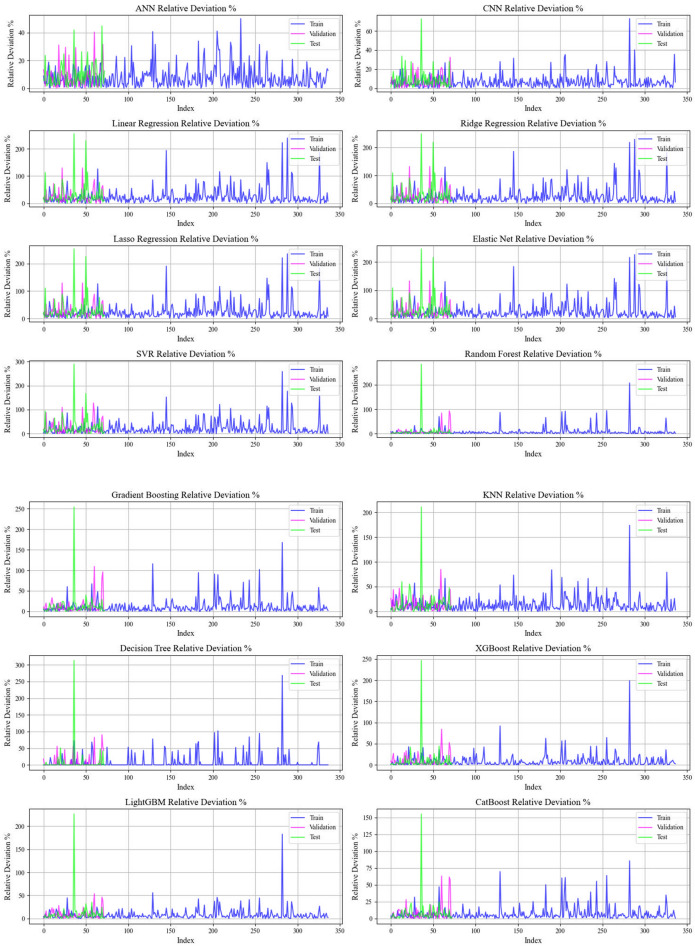
A comprehensive analysis of relative deviation percentages provided for all models employed in biomass yield prediction

**Fig. 10:**
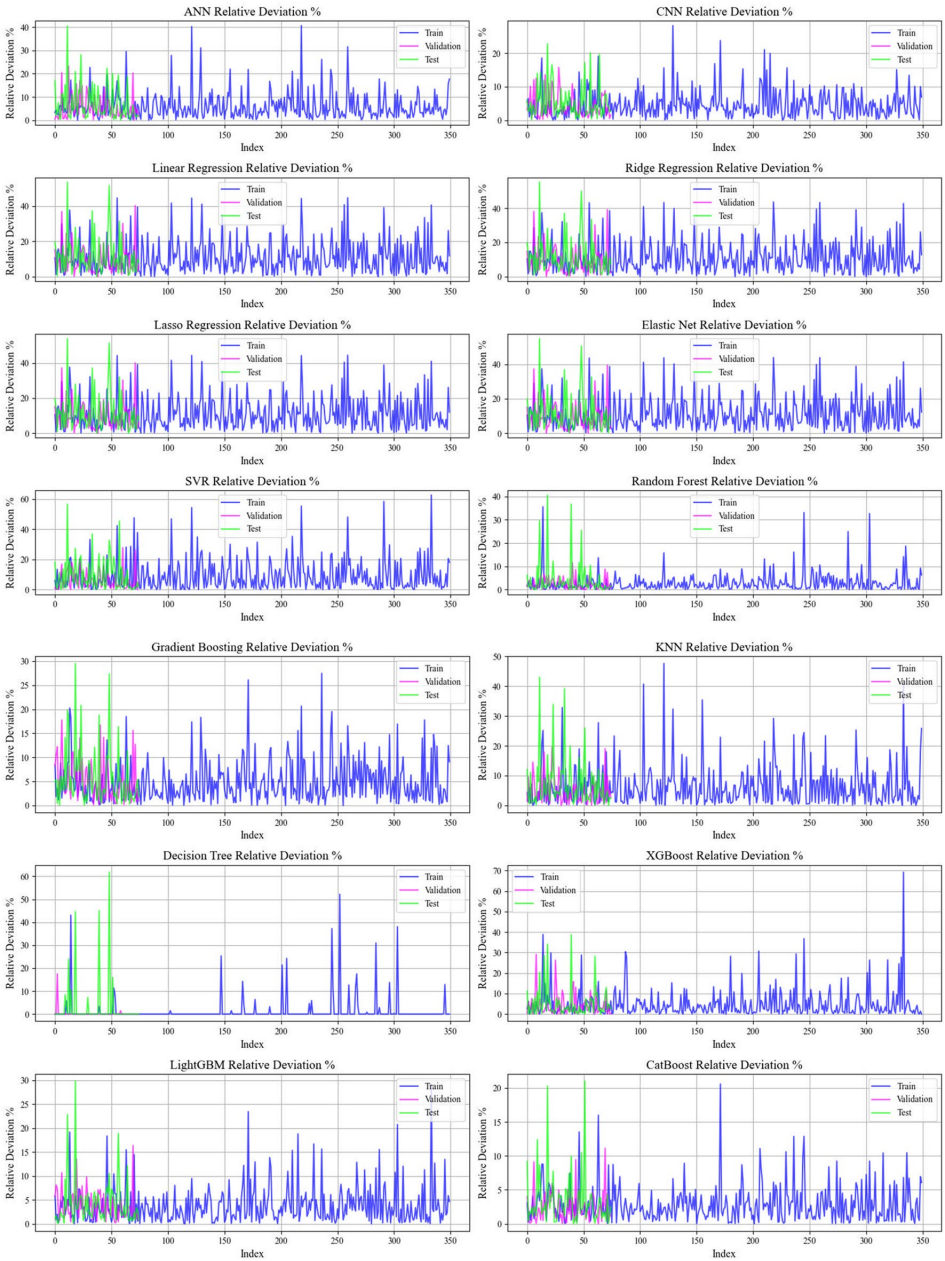
Relative deviation percentages meticulously detailed for the cohorts across the entire models employed for biomass yield prediction

Figures 9 and 10 explore error distributions over scatter plot of relative errors in predicting HHV and yield. In the case of the CatBoost model, the errors are evenly spread nearby the x-axis, signifying minimal variance and reliable prediction accuracy. This further reinforces the reliability of the model, enhancing confidence in its forecasting ability.

**Fig. 11:**
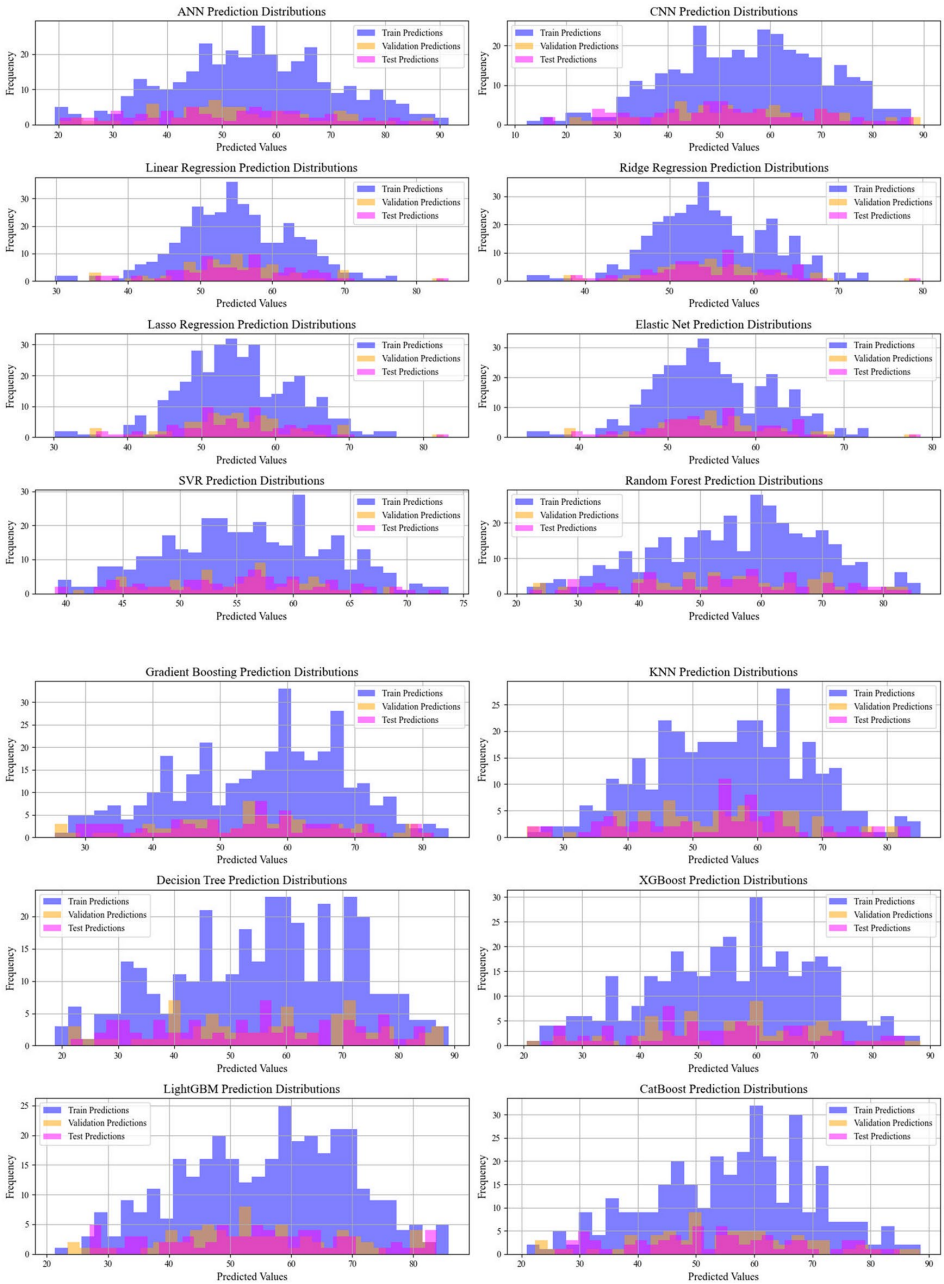
Information on the frequency of the data sets employed for biomass yield

**Fig. 12:**
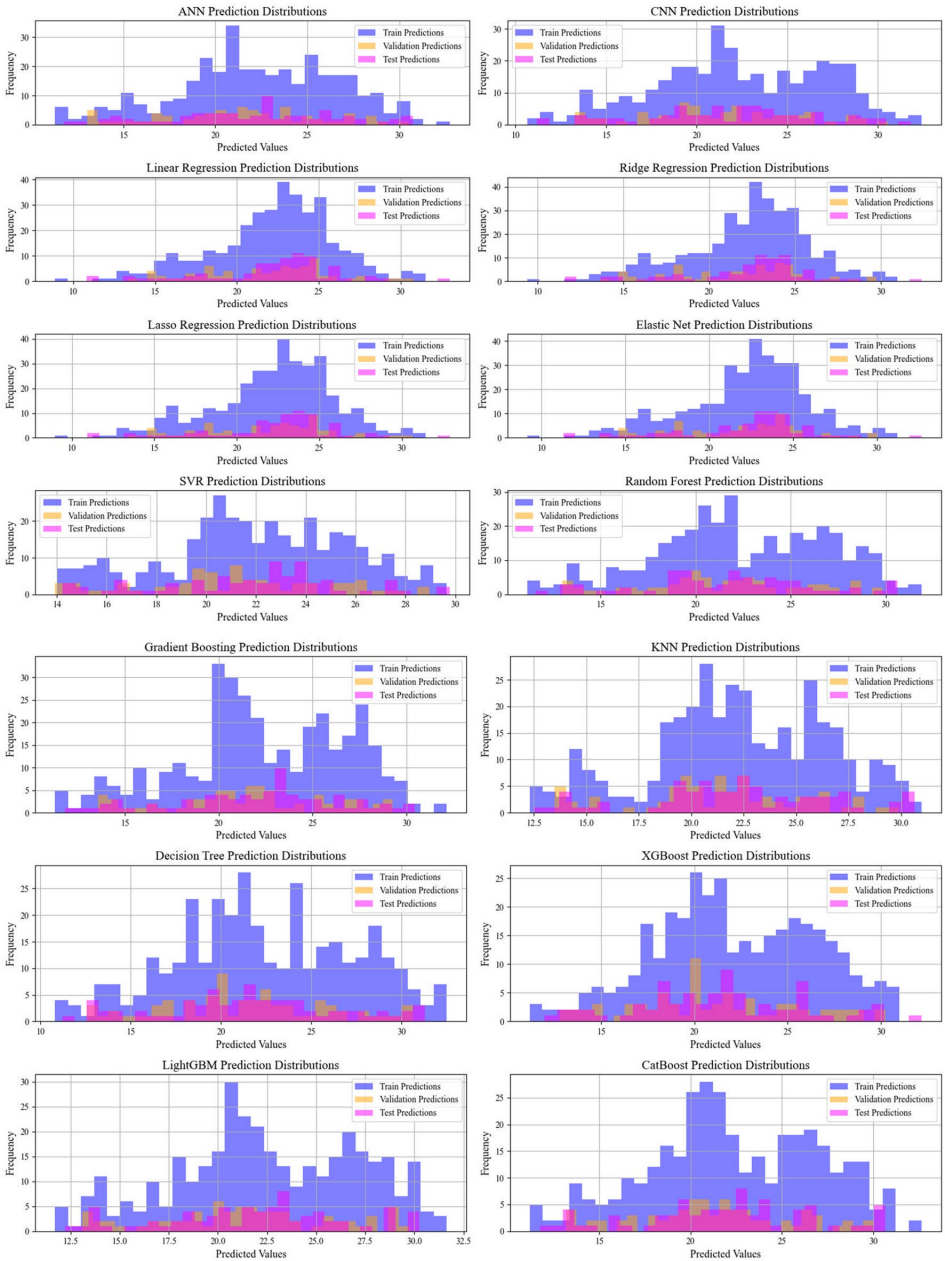
Information on the frequency of the data sets employed for biomass HHV prediction

Lastly, Figs. 11 and 12 illustrate the prediction distribution of all models across the different modeling phases for forcasing the yield and HHV. The performance of models in predicting HHV is evaluated by comparing the models’ outputs with empirical observations.This consistency highlights CatBoost’s robustness and stability, positioning it as the most reliable model for real-world usages.

To truly understand how machine learning models make predictions, it’s essential to know the significance of each input feature. In this research, we utilize Shapley Additive exPlanations (SHAP). This powerful method, rooted in game-theoretic principles, provides a clear and rigorous framework for interpreting model outputs, revealing precisely how individual features contribute to predictions.

**Fig. 13:**
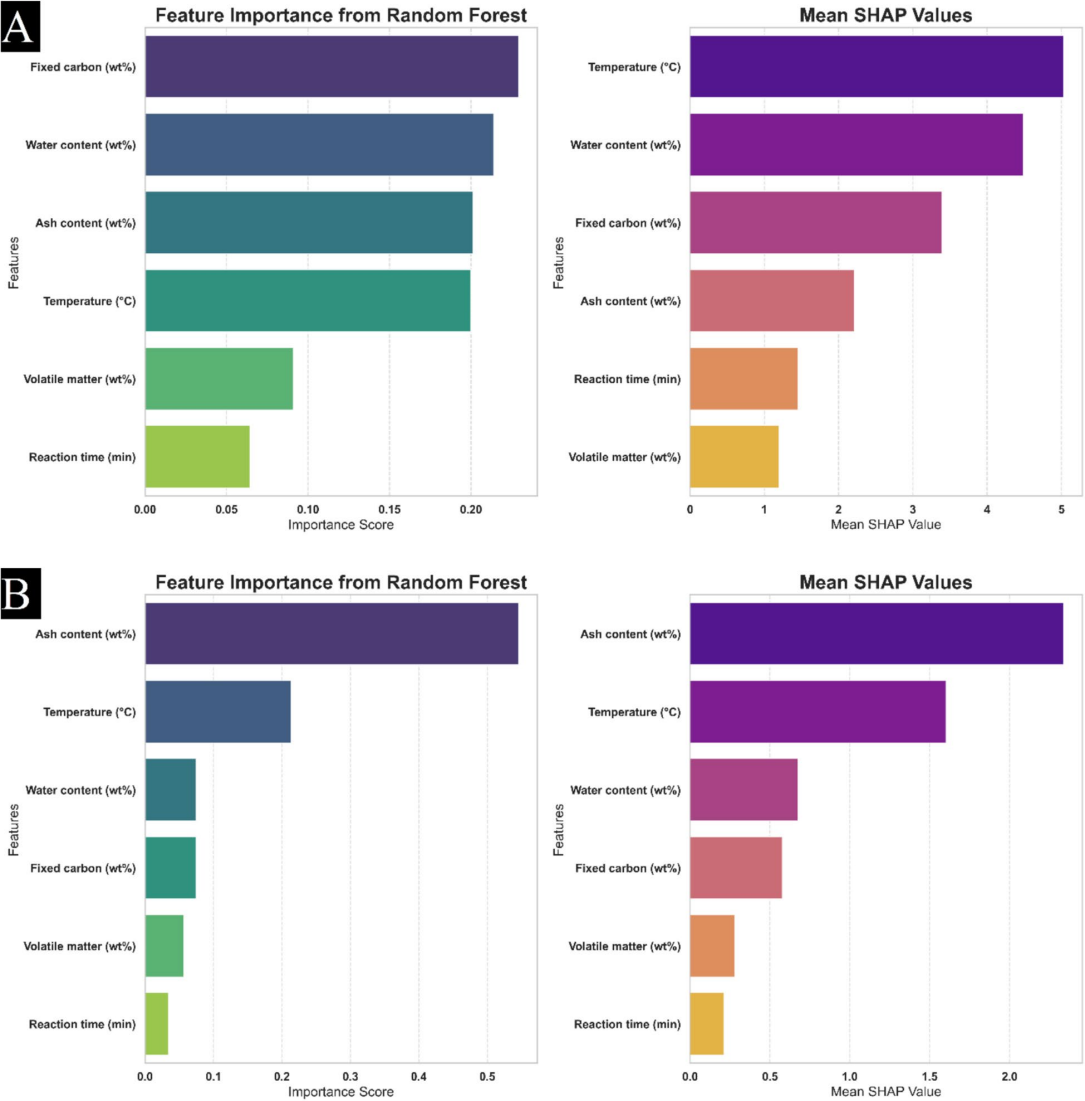
Random Forest and Mean SHAP Insights into feature contributions for prediction **A** yield and **B** HHV parameters

Figure 13A, B display the SHAP results for the inputs, along with the feature status derived from the Random Forest for calculating hydrochar HHV and yield regarding biomass proximate. The parameters are ranked in downward order according to their SHAP amounts, with the highest-ranked features exerting the most significant influence on model results. The investigation indicates that ash content is the primary determinant for HHV prediction, while temperature, water content, and fixed carbon are identified as the most crucial parameters for predicting yield.

These findings are instrumental in identifying the key factors that affect hydrochar HHV and yield in the context of biomass proximate analysis. By providing intuitions into the relationships between inputs and desired variables, better performance is facilitated, enhancing its utility in real-world industries. Additionally, clarity regarding feature significance is essential for guiding future research and optimization strategies, enabling decisions by quantifying the unique influence of each parameter on predictions.

The predictive power of our machine learning models stems from the inherent relationships between the proximate analysis of the raw biomass and the fundamental chemical and physical changes occurring during hydrothermal carbonization (HTC). A deeper mechanistic understanding of these relationships is essential for a more complete interpretation of our model’s predictions.

The fixed carbon (FC) content of the biomass is a key parameter directly influencing the final HHV of the hydrochar. Fixed carbon represents the non-volatile combustible material left after the release of volatile matter. During the HTC process, while some devolatilization occurs, the fixed carbon fraction remains largely intact. As a result, biomass with a higher initial FC content will naturally produce a hydrochar with a greater proportion of fixed carbon, thereby increasing its energy density and resulting in a higher HHV. Our models effectively capture this direct correlation, demonstrating that a higher FC content in the feedstock is a strong positive predictor for the final hydrochar HHV.

Conversely, the volatile matter (VM) content is a primary factor governing the final hydrochar yield. Volatile matter consists of organic compounds that are released as gases and liquids during the HTC process. This release of VM from the solid biomass matrix represents a significant mass loss. Consequently, biomass feedstocks with higher volatile matter content will undergo a greater degree of devolatilization and decomposition, leading to a lower overall hydrochar yield. Our models’ ability to predict yield based on VM content highlights the critical role of this parameter in determining the efficiency of solid fuel recovery during HTC.

Finally, the ash content and moisture content of the biomass also play important, albeit different, roles. Ash is an inert component that does not contribute to the combustion process. Therefore, a higher ash content in the feedstock dilutes the combustible fixed carbon and volatile matter in the resulting hydrochar, which invariably lowers its HHV. This effect is consistently captured by our models, reinforcing the importance of using low-ash biomass for high-quality hydrochar production. The initial moisture content acts as the reaction medium for the HTC process. While not a direct component of the final hydrochar’s chemical makeup, it influences the reaction kinetics, including the temperature and pressure profiles. These conditions affect the extent of carbonization and dehydration reactions, ultimately having a secondary but significant impact on both the hydrochar yield and HHV. The success of our models in predicting these properties from moisture content suggests that the models have learned these complex, indirect relationships.

This mechanistic analysis validates our models’ predictive capabilities and provides practical insights for optimizing the hydrochar production process. By controlling the input biomass’s proximate analysis, particularly by selecting feedstocks with high fixed carbon, low volatile matter, and low ash content, it is possible to produce a high-yield, high-HHV hydrochar, thereby maximizing the efficiency of this sustainable energy conversion pathway.

## Conclusion

This research delivers a inclusive investigation of biomass proximate analysis for forecasting hydrochar-yield and higher-heating-value (HHV) by machine learning. Key parameters such as fixed carbon, temperature,volatile matter, ash content, reaction time, and water content were analyzed, with Pearson correlation revealing their influence on hydrochar yield and HHV. The Monte Carlo Outlier Detecting (MCOD) ensured data reliability, enhancing the success of the modeling. Among the models evaluated, CatBoost outperformed others with R^2^ values of 0.90 (yield prediction) and 0.93 (HHV prediction), as well as low mean relative deviation (MRD) and mean squared error (MSE). The study highpoints the superiority of gradient-boosting methods over simpler models in capturing complex relationships. Visual validation tools further confirmed CatBoost’s accuracy and consistency, while SHAP analysis identified ash content as the key factor in HHV prediction and temperature, water content, and fixed carbon as critical for yield prediction. In conclusion, this research emphasizes the potential of advanced machine learning models, particularly gradient-boosting algorithms like CatBoost, in optimizing biomass-to-hydrochar conversion processes. These findings hold significant implications for improving bioenergy production and guiding future research in biomass modeling and conversion.

## Supplementary Information

Additional file1.

## Data Availability

Data is available in the supplementary material file.
